# pathfindR: An R Package for Comprehensive Identification of Enriched Pathways in Omics Data Through Active Subnetworks

**DOI:** 10.3389/fgene.2019.00858

**Published:** 2019-09-25

**Authors:** Ege Ulgen, Ozan Ozisik, Osman Ugur Sezerman

**Affiliations:** ^1^Department of Biostatistics and Medical Informatics, School of Medicine, Acibadem Mehmet Ali Aydinlar University, Istanbul, Turkey; ^2^Department of Computer Engineering, Electrical & Electronics Faculty, Yildiz Technical University, Istanbul, Turkey

**Keywords:** pathway analysis, enrichment, tool, active subnetworks, biological interaction network

## Abstract

Pathway analysis is often the first choice for studying the mechanisms underlying a phenotype. However, conventional methods for pathway analysis do not take into account complex protein-protein interaction information, resulting in incomplete conclusions. Previously, numerous approaches that utilize protein-protein interaction information to enhance pathway analysis yielded superior results compared to conventional methods. Hereby, we present pathfindR, another approach exploiting protein-protein interaction information and the first R package for active-subnetwork-oriented pathway enrichment analyses for class comparison omics experiments. Using the list of genes obtained from an omics experiment comparing two groups of samples, pathfindR identifies active subnetworks in a protein-protein interaction network. It then performs pathway enrichment analyses on these identified subnetworks. To further reduce the complexity, it provides functionality for clustering the resulting pathways. Moreover, through a scoring function, the overall activity of each pathway in each sample can be estimated. We illustrate the capabilities of our pathway analysis method on three gene expression datasets and compare our results with those obtained from three popular pathway analysis tools. The results demonstrate that literature-supported disease-related pathways ranked higher in our approach compared to the others. Moreover, pathfindR identified additional pathways relevant to the conditions that were not identified by other tools, including pathways named after the conditions.

## Introduction

High-throughput technologies revolutionized biomedical research by enabling comprehensive characterization of biological systems. One of the most common use cases of these technologies is to perform experiments comparing two groups of samples (typically disease versus control) and identify a list of altered genes. However, this list alone often falls short of providing mechanistic insights into the underlying biology of the disease being studied (Khatri et al., 2012). Therefore, researchers face a challenge posed by high-throughput experiments: extracting relevant information that allows them to understand the underlying mechanisms from a long list of genes.

One approach that reduces the complexity of analysis while simultaneously providing great explanatory power is identifying groups of genes that function in the same pathways, i.e., pathway analysis. Pathway analysis has been successfully and repeatedly applied to gene expression (Werner, 2008; Emmert-Streib and Glazko, 2011), proteomics (Wu et al., 2014), and DNA methylation data (Wang et al., 2017).

Most commonly used pathway analysis methods are overrepresentation analysis (ORA) and functional class scoring (FCS). For each pathway, ORA statistically evaluates the proportion of altered genes among the pathway genes against the proportion among a set of background genes. In FCS, a gene-level statistic is calculated using the measurements from the experiment. These gene-level statistics are then aggregated into a pathway-level statistic for each pathway. Finally, the significance of each pathway-level statistic is assessed, and significant pathways are determined.

While they are widely used, there are drawbacks to conventional pathway analysis methods. The statistics used by ORA approaches usually consider the number of genes in a list alone. ORA methods are also independent of the values associated with these genes, such as fold changes or p values. Most importantly, both ORA and FCS methods lack in incorporating interaction information. We propose that directly performing pathway analysis on a gene set is not completely informative because this approach reduces gene-phenotype association evidence by ignoring information on interactions of genes.

We propose a pathway analysis method, which we named pathfindR, that first identifies active subnetworks and then performs enrichment analysis using the identified active subnetworks. For a given list of significantly altered genes, an active subnetwork is defined as a group of interconnected genes in a protein-protein interaction network (PIN) that predominantly consists of significantly altered genes. In other words, active subnetworks define distinct disease-associated sets of interacting genes. 

The idea of utilizing PIN information to enhance pathway enrichment results was sought and successfully implemented in numerous studies. Gene Network Enrichment Analysis (GNEA) (Liu et al., 2007) analyzes gene expression data. The mRNA expression of every gene is mapped onto a PIN, and a significantly transcriptionally affected subnetwork is identified via jActiveModules (Ideker et al., 2002). To determine the gene set enrichment, each gene set is then tested for overrepresentation in the subnetwork. In EnrichNet (Glaab et al., 2012), input genes and pathway genes are mapped on a PIN. Using the random walk with restart (RWR) algorithm, distances between input genes and pathway genes are calculated. Enrichment results are obtained by comparing these distances to a background model. In both NetPEA and NetPEA′ (Liu et al., 2017a), initially, the RWR algorithm is used to measure distances between pathways and input gene sets. The significances of pathways are then calculated by comparing against a background model created with two different approaches: a) randomizing input genes (NetPEA) and b) randomizing input genes and the PIN (NetPEA′).

With pathfindR, our aim was likewise to exploit interaction information to extract the most relevant pathways. We aimed to combine together active subnetwork search and pathway enrichment analysis. By implementing this original active-subnetwork-oriented pathway analysis approach as an R package, our intention was to provide the research community with a set of utilities (in addition to pathway analysis, clustering of pathways, scoring of pathways, and visualization utilities) that will be effective, beneficial, and straightforward to utilize for pathway enrichment analysis exploiting interaction information.

The active-subnetwork-oriented pathway enrichment paradigm of pathfindR can be summarized as follows: Mapping the statistical significance of each gene onto a PIN, active subnetworks, i.e., subnetworks in the PIN that contain an optimal number of significant nodes maximizing the overall significance of the subnetwork, either in direct contact or in indirect contact via an insignificant (non-input) node, are identified. Following a subnetwork filtering step, enrichment analyses are then performed on these active subnetworks. Similar to the above-mentioned PIN-aided enrichment approaches, utilization of active subnetworks allows for efficient exploitation of interaction information and enhances enrichment analysis.

For the identification of active subnetworks, various algorithms have been proposed, such as greedy algorithms (Breitling et al., 2004; Sohler et al., 2004; Chuang et al., 2007; Nacu et al., 2007; Ulitsky and Shamir, 2007; Karni et al., 2009; Ulitsky and Shamir, 2009; Fortney et al., 2010; Doungpan et al., 2016), simulated annealing (Ideker et al., 2002; Guo et al., 2007), genetic algorithms (Klammer et al., 2010; Ma et al., 2011; Wu et al., 2011; Amgalan and Lee, 2014; Ozisik et al., 2017), and mathematical programming-based methods (Dittrich et al., 2008; Zhao et al., 2008; Qiu et al., 2009; Backes et al., 2012; Beisser et al., 2012). In pathfindR, we provide implementations for a greedy algorithm, a simulated annealing algorithm, and a genetic algorithm.

In summary, pathfindR integrates information from three main resources to enhance determination of the mechanisms underlying a phenotype: (i) differential expression/methylation information obtained through omics analyses, (ii) interaction information through a PIN via active subnetwork identification, and (iii) pathway/gene set annotations from sources such as Kyoto Encyclopedia of Genes and Genomes (KEGG) (Kanehisa and Goto, 2000; Kanehisa et al., 2017), Reactome (Fabregat et al., 2018), BioCarta (Nishimura, 2001), and Gene Ontology (GO) (Ashburner et al., 2000).

The pathfindR R (https://www.R-project.org/) package was developed based on a previous approach developed by our group for genome-wide association studies (GWASes): Pathway and Network-Oriented GWAS Analysis (PANOGA) (Bakir-Gungor et al., 2014). PANOGA was successfully applied to uncover the underlying mechanisms in GWASes of various diseases, such as intracranial aneurysm (Bakir-Gungor and Sezerman, 2013), epilepsy (Bakir-Gungor et al., 2013), and Behcet’s disease (Bakir-Gungor et al., 2015). With pathfindR, we aimed to extend the approach of PANOGA to omics analyses and provide novel functionality.

In this article, we present an overview of pathfindR, example applications on three gene expression data sets, and comparison of the results of pathfindR with those obtained using three tools widely used for enrichment analyses: The Database for Annotation, Visualization and Integrated Discovery (DAVID) (Huang da et al., 2009), Signaling Pathway Impact Analysis (SPIA) (Tarca et al., 2009), and Gene Set Enrichment Analysis (GSEA) (Subramanian et al., 2005).

## Material and Methods

### PINs and Gene Sets

PIN data available in pathfindR by default are KEGG, Biogrid (Stark et al., 2006; Chatr-Aryamontri et al., 2017), GeneMania (Warde-Farley et al., 2010), and IntAct (Orchard et al., 2014). The default PIN is Biogrid. Besides these four default PINs, the researcher can also use any other PIN of their choice on the condition that they provide the PIN file in simple interaction file (SIF) format.

The KEGG Homo sapiens PIN was created by an in-house script using the KEGG pathways. In KEGG, pathways are represented in XML files that contain genes and gene groups, such as protein complexes as entries and interactions as entry pairs. The KEGG pathway XML files were obtained using the official KEGG Application Programming Interface (API) which is a REST-style interface to the KEGG database resource. Using the in-house script, the XML files were parsed; the interactions were added as undirected pairs, while interaction types were disregarded. In cases of an entry in an interacting pair containing multiple genes, interactions from all of these genes to the other entry were built.

For Biogrid, Homo sapiens PIN data in tab-delimited text format from release 3.4.156 (BIOGRID-ORGANISM-Homo_sapiens-3.4.156.tab.txt) was obtained from the Biogrid Download File Repository (https://downloads.thebiogrid.org/BioGRID). 

For IntAct, the PIN data in Proteomics Standards Initiative – Molecular Interactions tab-delimited (PSI-MI TAB) (MITAB) format (intact.txt) were obtained from the IntAct Molecular Interaction Database FTP site (ftp://ftp.ebi.ac.uk/pub/databases/intact/current) in January 2018. 

For GeneMania, Homo sapiens PIN data in tab-delimited text format from the latest release (COMBINED.DEFAULT_NETWORKS.BP_COMBINING.txt) was obtained from the official data repository (http://genemania.org/data/current/Homo_sapiens.COMBINED/). For this PIN only, only interactions with GeneMania weights ≥0.0006 were kept, allowing only strong interactions.

No filtration for interaction types were performed for any PIN (i.e., all types of interactions were kept). The processing steps performed for all the PINs were (1.) if the HUGO Gene Nomenclature Committee (HGNC) symbols for interacting genes were not provided, conversion of provided gene identifiers to HGNC symbols using biomaRt (Durinck et al., 2009) was performed; (2.) duplicate interactions and self-interactions (if any) were removed; and (3.) all PINs were formatted as SIFs.

Gene sets available in pathfindR are KEGG, Reactome, BioCarta, GO-Biological Process (GO-BP), GO-Cellular Component (GO-CC), GO-Molecular Function (GO-MF) and GO-All (GO-BP, GO-CC, and GO-MF combined).

KEGG gene sets were obtained using the R package KEGGREST. Reactome gene sets in Gene Matrix Transposed (GMT) file format were obtained from the Reactome website (https://reactome.org/download/current/). BioCarta gene sets in GMT format were retrieved from the Molecular Signatures Database (MSigDB) (Liberzon et al., 2011) website (http://software.broadinstitute.org/gsea/msigdb). All “High-quality” GO gene sets were obtained from GO2MSIG (Powell, 2014) web interface (http://www.go2msig.org/cgi-bin/prebuilt.cgi?taxid=9606) in GMT format. All of the datasets were processed using R to obtain (1) a list containing the genes involved in each given gene set/pathway (hence, each element of the list is named by the gene set ID and is a vector of gene symbols located in the given gene set/pathway) and (2) a list containing the descriptions for each gene set/pathway (i.e., a list linking gene set IDs to description).

All of the gene sets in pathfindR are for Homo sapiens, and the default gene set is KEGG. The researcher can also use a gene set of their choice following the instructions on pathfindR wiki.

All of the default data for PINs and gene sets are planned to be updated annually.

### Scoring of Subnetworks

In pathfindR, we followed the scoring scheme that was proposed by Ideker et al., 2002). The p value of each gene is converted to a z score using equation (1), and the score of a subnetwork is calculated using equation (2). In equation (1) Φ^–1^ is the inverse normal cumulative distribution function. In equation (2), A is the set of genes in the subnetwork and k is its cardinality.

(1) $$z_{i}=\Phi^{-1}(1-p_{i})$$

(2) $$z_{A}=\frac{1}{\sqrt{k}}\sum_{i\in A}z_{i}$$

In the same scoring scheme, a Monte Carlo approach is used for the calibration of the scores of subnetworks against a background distribution. Using randomly selected genes, 2,000 subnetworks of each possible size are constructed, and for each possible size, the mean and standard deviation of the score is calculated. These values are used to calibrate the subnetwork score using equation (3).

(3) $$s_{A}=\frac{\left(z_{A}-\mu_{k}\right)}{\sigma_{k}}$$

### Active Subnetwork Search Algorithms

Currently, there are three algorithms implemented in the pathfindR package for active subnetwork search, described below.

#### Greedy Algorithm

Greedy algorithm is the problem-solving/optimization concept that chooses locally the best option in each stage with the expectation of reaching the global optimum. In active subnetwork search, this is generally applied by starting with a significant seed node and considering addition of a neighbor in each step to maximize the subnetwork score. In pathfindR, we used the approach described by Chuang et al. (2007). This algorithm considers addition of a node within a specified distance d to the current subnetwork. In our method, the maximum depth from the seed can also be set. With the default parameters, our greedy method considers addition of direct neighbors (d = 1) and forms a subnetwork with a maximum depth of 1 for each seed. Because the expansion process runs for each significant seed node, several overlapping subnetworks emerge. Overlapping subnetworks are handled by discarding a subnetwork that overlaps with a higher scoring subnetwork more than a given threshold, which is set to 0.5 by default.

#### Simulated Annealing Algorithm

Simulated annealing is an optimization algorithm inspired by annealing in metallurgy. In the annealing process, the material is heated above its recrystallization temperature and cooled slowly, allowing atoms to diffuse within the material and decrease dislocations. Analogous to this process, simulated annealing algorithm starts with a “high temperature” in which there is a high probability of accepting a solution that is worse than the current one as the solution space is explored. The acceptability of worse solutions allows a global search and escaping from local optima. The equation connecting temperature and probability of accepting a new solution is given in equation (4). In this equation, P(Acceptance) is the probability of accepting the new solution. In score_new_ and score_current_ are the scores of the new and the current solutions, respectively. Finally, temperature is the current temperature.

(4) $$P\left(\mathrm{Acceptance}\right)=\;\{\begin{array}{c} 1,\mathrm{if}\;\mathrm{Scor}e_{\mathrm{new}}-\;\mathrm{Scor}e_{\text{current}}>0\\ e^{\frac{\mathrm{Scor}e_{\mathrm{new}}-\;\mathrm{Scor}e_{\mathrm{current}}}{\mathrm{temperature}}},\;\;\mathrm{otherwise} \end{array}$$

A less worse solution and higher temperature are the conditions that increase the chance of acceptation of a new solution. The probability of accepting a non-optimal action decreases in each iteration, as the temperature decreases in each step.

Simulated annealing provides improved performance over the greedy search by accepting non-optimal actions to increase exploration in the search space. In the active subnetwork search context, the search begins with a set of randomly chosen genes (the chosen genes are referred to as genes in “on” state and the not chosen genes are referred to as genes in “off” state). Connected components in this candidate solution are found, and the scores are calculated. In each iteration, the state of a random node is changed from on to off and vice versa. Connected components are found in the new solution, and their scores are calculated. If the score improves, the change is accepted. If the score decreases, the change is accepted with a probability proportional to the temperature parameter that decreases in each step.

#### Genetic Algorithm

Genetic algorithm is a bio-inspired algorithm that mimics evolution by implementing natural selection, chromosomal crossover, and mutation. The main phases of the genetic algorithm are “the selection phase” and “the crossover phase.”

In the selection phase, parents from the existing population are selected through a fitness-based process to breed a new generation. Common selection methods are (i) roulette wheel selection in which a solution’s selection probability is proportional to its fitness score, (ii) rank selection in which a solution’s selection probability is proportional to its rank, thus preventing the domination of a high fitness solution to the rest, and (iii) tournament selection in which parents are selected among the members of randomly selected groups of solutions, thus giving more chance to small fitness solutions that would have little chance in other selection methods.

In the crossover phase, encoded solution parameters of the parents are exchanged analogous to chromosomal crossover. The common crossover operators are (i) single-point crossover in which the segment next to a randomly chosen point in the solution representation is substituted between parents, (ii) two-point crossover in which the segment between two randomly chosen points is substituted, and (iii) uniform crossover in which each parameter is randomly selected from either of the parents. Mutation is the process of randomly changing parameters in the offspring solutions in order to maintain genetic diversity and explore search space.

In our genetic algorithm implementation, candidate solutions represent the on/off state of each gene. In the implementation, we used rank selection and uniform crossover. In each iteration, the fittest solution of the previous population is preserved if the highest score of the current population is less than the previous population’s score. In every 10 iterations, the worst scoring 10% of the population is replaced with random solutions. Because uniform cross-over and addition of random solutions make adequate contribution to the exploration of the search space, mutation is not performed under the default settings.

#### Selecting the Active Subnetwork Search Algorithm

The default search method in pathfindR is greedy algorithm with a search depth of 1 and maximum depth of 1. This method stands out with its simplicity and speed. This is also the “local subnetwork approach” used in the Local Enrichment Analysis (LEAN) method (Gwinner et al., 2017). As mentioned in the LEAN study, the number of subnetworks to be identified typically increases exponentially with increasing number of genes in the PIN, and the “local subnetwork approach” enables iterating over each local subnetwork and determining phenotype-related clusters. Greedy algorithm with search depth and maximum depth equal to 2 or more lets the search algorithm look further in the network for another significant gene to add to the cluster, but this may result in a slower runtime and a loss in interpretability.

Simulated annealing and genetic algorithms are heuristic methods that do not make any assumptions on the active subnetwork model. They can let insignificant genes between two clusters of significant genes to create a single connected active subnetwork. Thus, these algorithms may result in a large highest scoring active subnetwork, while the remaining subnetworks identified become small and therefore uninformative. This tendency towards large subnetworks was attributed to a statistical bias prevalent in many tools (Nikolayeva et al., 2018).

The default active search method (greedy algorithm with a search depth of 1 and maximum depth of 1) in pathfindR was preferred because multiple active subnetworks are used for enrichment analyses. If the researcher decides to use the single highest scoring active subnetwork for the enrichment process, they are encouraged to consider greedy algorithm with greater depth, simulated annealing, or genetic algorithm.

### Active-Subnetwork-Oriented Pathway Enrichment Analysis

**Figure 1:**
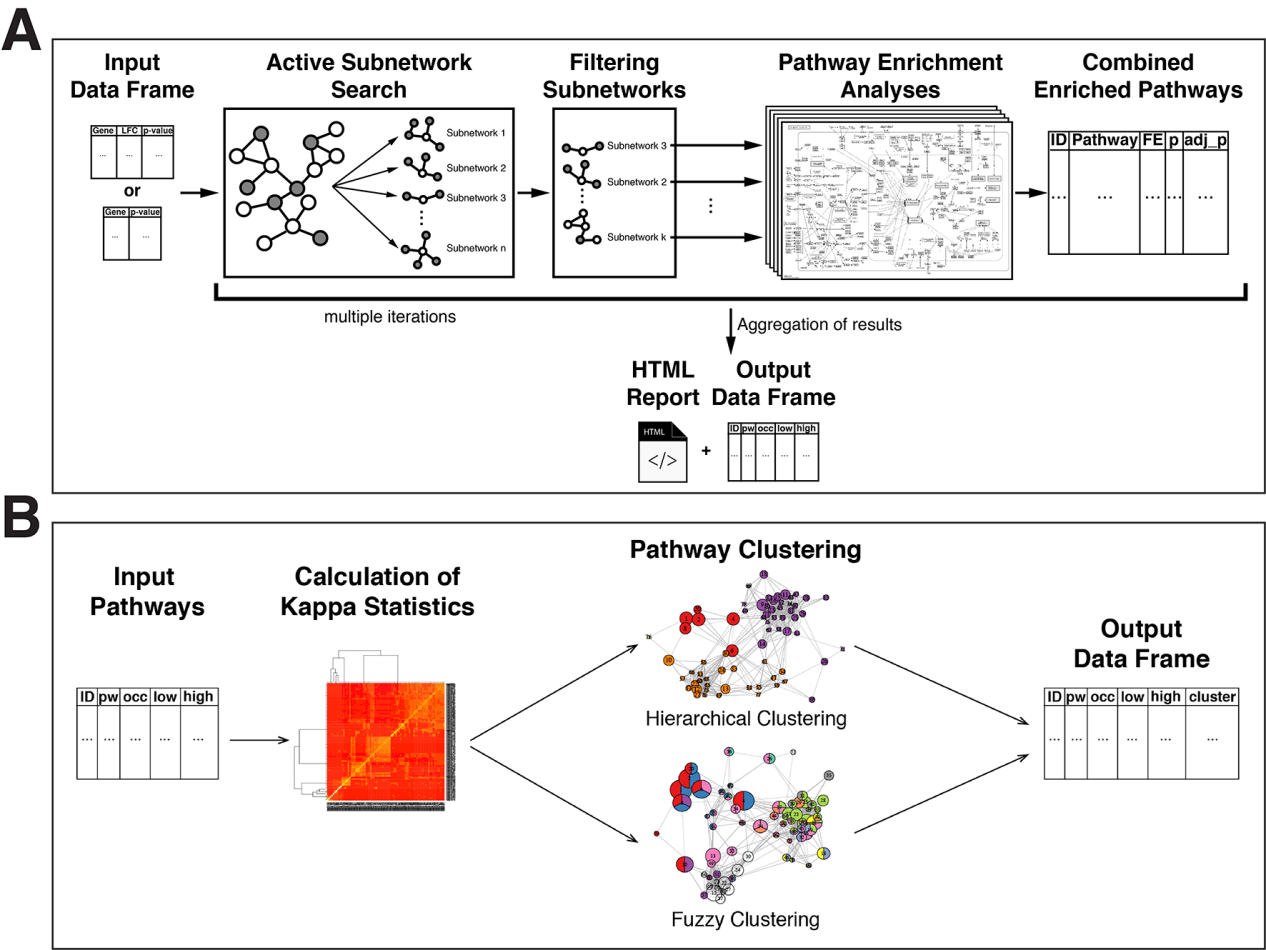
Flow diagrams of the pathfindR methods. **(A)** Flow diagram of the pathfindR active-subnetwork-oriented pathway enrichment analysis approach. **(B)** Flow diagram of the pathfindR pathway clustering approaches.

The overview of the active-subnetwork-oriented pathway enrichment approach is presented in **Figure 1A**.

The required input is a two- or three-column table: Gene symbols, change values as log-fold change (optional) and adjusted p values associated with the differential expression/methylation data.

Initially, the input is filtered so that all p values are less than or equal to the given threshold (default is 0.05). Next, gene symbols that are not in the PIN are identified. If aliases of these gene symbols are found in the PIN, these symbols are converted to the corresponding aliases. 

The processed data are then used for active subnetwork search. The identified active subnetworks are filtered via the following criteria: (i) has a score larger than the given quantile threshold (default is 0.80) and (ii) contains at least a specified number of input genes (default is 10).

For each filtered active subnetwork, using the genes contained in each of these subnetworks, separate pathway enrichment analyses are performed via one-sided hypergeometric testing. The enrichment tests use the genes in the PIN as the gene pool (i.e., background genes). Using the genes in the PIN instead of the whole genome is more appropriate and provides more statistical strength because active subnetworks are identified using only the genes in the PIN. Next, the p values obtained from the enrichment tests are adjusted (default is by Bonferroni method. However, the researcher may choose another method they prefer). Pathways with adjusted p values larger than the given threshold (default is 0.05) are discarded. These significantly enriched pathways per all filtered subnetworks are then aggregated by keeping only the lowest adjusted p value for each pathway if a pathway was found to be significantly enriched in the enrichment analysis of more than one subnetwork.

This process of active subnetwork search and enrichment analysis (active subnetwork search, filtering of subnetworks, enrichment analysis on each filtered subnetwork, and aggregation of enrichment results over all subnetworks) is repeated for a selected number of iterations (default is 10 iterations for greedy and simulated annealing algorithms, 1 for genetic algorithm).

Finally, the lowest and the highest adjusted p values, the number of occurrences over all iterations, and up-regulated and down-regulated genes in each enriched pathway are returned as a table. Additionally, a Hypertext Markup Language (HTML) format report with the pathfindR enrichment results is created. Pathways are linked to the visualizations of the pathways if KEGG gene sets are chosen. The KEGG pathway diagrams are created using the R package pathview (Luo and Brouwer, 2013). By default, these diagrams display the involved genes colored by change values, normalized between −1 and 1, on a KEGG pathway graph. If a gene set other than KEGG is chosen and visualization is required, graphs of interactions of genes involved in the enriched pathways in the chosen PIN are visualized via the R package igraph (Csardi and Nepusz, 2006).

### Pathway Clustering

Enrichment analysis usually yields a large number of related pathways. In order to establish representative pathways among similar groups of pathways, we propose that clustering can be performed either via hierarchical clustering (default) or via a fuzzy clustering method as described by Huang et al. (2007). These clustering approaches are visually outlined in **Figure 1B** and described below:

Firstly, using the input genes in each pathway, a kappa statistics matrix containing the pairwise kappa statistics, a chance-corrected measure of co-occurrence between two sets of categorized data, between the pathways is calculated (Huang et al., 2007).

By default, the wrapper function for pathway clustering, cluster_pathways, performs agglomerative hierarchical clustering (defining the distance as 1 − kappa statistic), automatically determines the optimal number of clusters by maximizing the average silhouette width, and returns a table of pathways with cluster assignments.

Alternatively, the fuzzy clustering method, previously proposed and described in detail by Huang et al. (2007), can be used to obtain fuzzy cluster assignments. Hence, this fuzzy approach allows a pathway to be a member of multiple clusters.

Finally, the representative pathway for each cluster is assigned as the pathway with the lowest adjusted p value.

### Pathway Scoring Per Sample

The researcher can get an overview of the alterations of genes in a pathway via the KEGG pathway graph. To provide even more insight into the activation/repression statuses of pathways per each sample, we devised a simple scoring scheme that aggregates gene-level values to pathway scores, described below.

For an experiment values matrix (e.g., gene expression values matrix), EM, where columns indicate samples and rows indicate genes, the gene score GS of a gene g in a sample s is calculated as:

(5) $$\mathrm{GS}\left(g,s\right)=\;\frac{EM_{g,s}-\;{\bar{X}}_{g}}{sd_{g}}$$

Here, ${\bar{X}}_{g}$ is the mean value for gene g across all samples, and *sd* *_g_* is the standard deviation for gene g across all samples.

For a set *P* *_i_*, the set of k genes in pathway *i*, and a sample* j*, the *i* ^th^ row and *j* ^th^ column of the pathway score matrix PS is calculated as follows:

(6) $$PS_{i,j}=\;\frac{1}{k}\sum_{g\in P_{i}}\mathrm{GS}\left(g,\;j\right)$$

The pathway score of a sample for a given pathway is therefore the average value of the scores of the genes in the pathway for the given sample.

After calculation of the pathway score matrix, a heat map of these scores is plotted. Via this heat map, the researcher can examine the activity of a pathway in individual samples as well as compare the overall activity of the pathway between cases and controls.

### Application on Gene Expression Datasets

To analyze the performance of pathfindR, we used three gene expression datasets. All datasets were obtained via the Gene Expression Omnibus (GEO) (Edgar et al., 2002). The first dataset (GSE15573) aimed to characterize and compare gene expression profiles in the peripheral blood mononuclear cells of 18 rheumatoid arthritis (RA) patients versus 15 healthy subjects using the Illumina human-6 v2.0 expression bead chip platform. This dataset will be referred to as RA. The second dataset (GSE4107) compared the gene expression profiles of the colonic mucosa of 12 early onset colorectal cancer patients and 10 healthy controls using the Affymetrix Human Genome U133 Plus 2.0 Array platform. The second dataset will be referred to as CRC. The third dataset (GSE55945) compared the expression profiles of prostate tissue from 13 prostate cancer patients versus 8 controls using the Affymetrix Human Genome U133 Plus 2.0 Array platform. This dataset will be referred to as PCa.

After preprocessing, which included log2 transformation and quantile normalization, differential expression testing via a moderated t test using limma (Ritchie et al., 2015) was performed. Next, the resulting p values were corrected using false discovery rate (FDR) adjustment. The differentially expressed genes (DEGs) were defined as those with FDR < 0.05. Probes mapping to multiple genes and probes that do not map to any gene were excluded. If a gene was targeted by multiple probes, the lowest p value was kept. The results of differential expression analyses for RA, CRC, and PCa, prior to filtering (differential expression statistics for all probes) and after filtering (lists of DEGs), are provided in **Supplementary Data Sheet 1**.

We chose to use these three datasets because these are well-studied diseases and the involved mechanisms are considerably well characterized. These different datasets also allowed us to test the capabilities of pathfindR on DEGs obtained from different platforms. 

We performed enrichment analysis with pathfindR, using the default settings. Greedy algorithm for active subnetwork search was used, and the analysis was carried out over 10 iterations. The enrichment significance cutoff value was set to 0.25 for each analysis (changing the argument enrichment_threshold of run_pathfindR function) as we later performed validation of the results using the three significance cutoff values of 0.05, 0.1, and 0.25

To better evaluate the performance of pathfindR, we compared results on the three gene expression datasets by three widely used pathway analysis tools, namely, DAVID (Huang da et al., 2009), SPIA (Tarca et al., 2009), and GSEA (Subramanian et al., 2005). DAVID 6.8 was used for the analyses. SPIA was performed using the default settings. GSEA was also performed using the default settings (using phenotype permutations). Additionally, pre-ranked GSEA was performed (GSEAPreranked) using the default settings. The rank of the *i* ^th^ gene *rank*_i_ was calculated as follows:

(7) $$\mathrm{ran}k_{i}=\;\{\begin{array}{c} -p_{i},\mathrm{if}\;\mathrm{logF}C_{i}<0\\ p_{i},\mathrm{otherwise} \end{array}$$

The unfiltered results of enrichment analyses using the different methods on the three datasets are presented in **Supplementary Data Sheet 2**.

For each analysis, the Bonferroni-corrected p values for pathfindR were used to filter the results. For all the other tools, as the Bonferroni method would be too strict and result in too few or no significant pathways, the FDR-corrected p values were used.

Because there is no definitive answer to which pathways are involved in the pathogenesis of the conditions under study, we analyzed the results in light of the existing biological knowledge on the conditions and compared our results with other tools in this context. The significant pathways were assessed on the basis of how well they fitted with the existing knowledge. For this, two separate approaches were taken: (i) assessment of literature support for the significantly enriched pathways (using a significance threshold of 0.05), and (ii) assessment of the percentages of pathway genes that are also known disease genes (using the three significance thresholds of 0.05, 0.10, and 0.25). While both assessments could be separately used to determine the “disease-relatedness” of a pathway, we chose to use them both as these are complementary measures: the former is a more subjective but a comprehensive measure of association, and the latter is a limited but a more objective measure of association. For determining the percentages of known disease genes in each significantly enriched pathway, two curated lists were used. For the RA dataset, mapped genes in the curated list of SNPs associated with RA was obtained from the NHGRI-EBI Catalog of published genome-wide association studies (GWAS Catalog, retrieved on 19.12.2018) (MacArthur et al., 2017). These genes will be referred to as “RA Genes.” For the CRC and PCa datasets, the “Cancer Gene Census” (CGC) genes from the Catalogue of Somatic Mutations in Cancer (COSMIC, http://cancer.sanger.ac.uk, retrieved on 19.12.2018) were used. These genes will be referred to as “CGC Genes.”

### Assessment Using Permuted Inputs

We performed pathfindR analyses using real and permuted data with different sizes to assess the number of enriched pathways identified in the permuted data against the actual data. For this assessment, the RA data was used. The analyses were performed on data subsets taken as the top 200, 300, 400, and 500 most significant DEGs as well as the complete list of 572 DEGs. For each input size, 100 separate pathfindR analyses were performed on both the actual input data and permuted data. While the real input data were kept unchanged, for the permuted data, a random permutation of genes (using the set of all genes available on the microarray platform) was carried out at each iteration over 100 analyses. Analyses with pathfindR were performed using the default settings described above.

The distributions of the number of enriched pathways for actual vs. permuted data were compared using Wilcoxon rank sum test.

### ORA Assessment of the Effect of DEGs Without Any Interactions

We performed ORA as implemented in pathfindR (as the “enrichment” function) to assess any effect of removing DEGs without any interactions on enrichment results. For this purpose, ORA were performed for (i) the full lists of DEGs for all datasets and (ii) the lists of DEGs that are found in the Biogrid PIN. As gene sets, KEGG pathways were used. As background genes, all of the genes in the Biogrid PIN were used for both analyses so that the results could be comparable. The enrichment p values were adjusted using the FDR method. Pathway enrichment was considered significant if FDR was <0.05.

### Assessment of the Effect of PINs on Enrichment Results

To analyze the effect of the chosen PIN on the enrichment results, we performed pathfindR analyses using the four PINs provided by default: the Biogrid, GeneMania, IntAct, and KEGGPINs. For these analyses, the default settings were used with the default active search algorithm (greedy) and the default gene sets (KEGG).

### Software Availability

The pathfindR package is freely available for use under MIT license: https://cran.r-project.org/package=pathfindR . The code of the pathfindR package is deposited in a GitHub repository (https://github.com/egeulgen/pathfindR) along with a detailed wiki, documenting the features of pathfindR in detail. Docker images for the latest stable version and the development version of pathfindR are deposited on Docker Hub (https://hub.docker.com/r/egeulgen/pathfindr) 

## Results

### The RA Dataset

**Figure 2:**
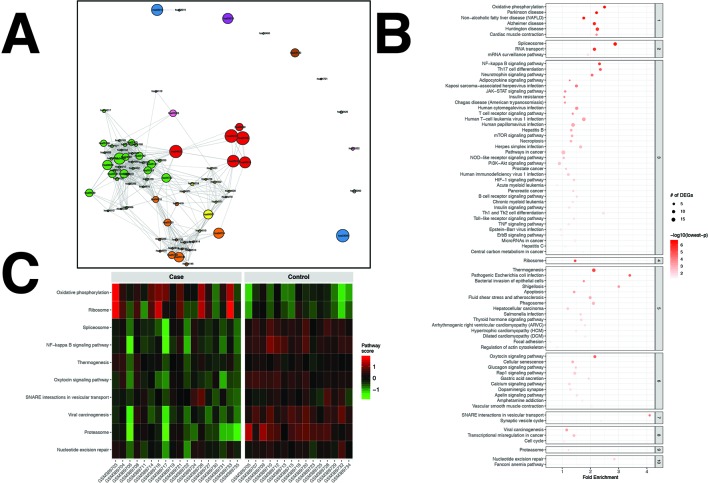
pathfindR enrichment and clustering results on the rheumatoid arthritis (RA) dataset (lowest p ≤ 0.05). **(A)** Clustering graph, each color displaying the clusters obtained for RA. Each node is an enriched pathway. Size of a node corresponds to its −log(lowest_p). The thickness of the edges between nodes corresponds to the kappa statistic between the two terms. **(B)** Bubble chart of enrichment results grouped by clusters (labeled on the right-hand side of each panel). The x axis corresponds to fold enrichment values, while the y axis indicates the enriched pathways. The size of the bubble indicates the number of differentially expressed genes (DEGs) in the given pathway. Color indicates the −log10(lowest-p) value; the more it shifts to red, the more significantly the pathway is enriched. **(C)** Heat map of pathway scores per subject. The x axis indicates subjects, whereas the y axis indicates representative pathways. Color scale for the pathway score is provided in the right-hand legend.

A total of 572 DEGs were identified for the RA dataset (**Supplementary Data Sheet 1**). Filtered by adjusted p values (adjusted-p ≤ 0.05), pathfindR identified 78 significantly enriched KEGG pathways which were partitioned into 10 clusters (**Figures 2A, B**). The relevancy of 31 out of 78 (39.74%) pathways was supported by literature, briefly stated in **Table 1**.

**Table 1:** Pathway analysis results for the rheumatoid arthritis (RA) dataset (adjusted p < 0.05).

ID	Pathway	% RA genes	pathfindR	DAVID	SPIA	GSEA	GSEAPreranked	Brief Description
hsa00190	Oxidative phosphorylation	0	**<0.001**	0.28157363	–	0.50656915	1	Oxygen metabolism has an important role in the pathogenesis of RA (Hitchon and El-Gabalawy, 2004; Yang et al., 2015).
hsa05012	Parkinson disease	1.41	**<0.001**	0.35202042	**0.03287527**	0.5198511	1	
hsa03040	Spliceosome	0	**<0.001**	0.19110635	–	–	–	Autoimmune response to the spliceosome was previously reported in numerous autoimmune diseases (Hassfeld et al., 1995).
hsa04932	Non-alcoholic fatty liver disease (NAFLD)	2.01	**<0.001**	–	–	–	–	
hsa05010	Alzheimer disease	0.58	**<0.001**	0.40188326	0.070524222	0.49685246	0.99091035	
hsa03013	RNA transport	0.61	**<0.001**	0.49158247	0.080862112	–	–	
hsa05016	Huntington disease	0.52	**<0.001**	0.24543866	0.03287527	0.5436461	1	
hsa04064	NF-kappa B signaling pathway	8.42	**<0.001**	0.634065	0.206122248	–	–	NF-kB is a pivotal mediator of inflammatory responses (Liu et al., 2017b) and an important player in RA pathogenesis (Makarov, 2001).
hsa03010	Ribosome	0	**<0.001**	–	–	0.6974111	–	
hsa04714	Thermogenesis	0.43	**<0.001**	–	–	–	–	
hsa05130	Pathogenic Escherichia coli infection	0	**<0.001**	0.42959791	0.103834432	0.740603	0.96458197	Possibly related to generation of neo-autoantigens, molecular mimicry, and bystander activation of the immune system (Li et al., 2013)
hsa04659	Th17 cell differentiation	19.63	**<0.001**	–	–	–	–	Th17 cells play an important role in inflammation in human autoimmune arthritides, including RA (Pernis, 2009; Leipe et al., 2010).
hsa04921	Oxytocin signaling pathway	1.97	**<0.001**	–	–	–	–	
hsa04722	Neurotrophin signaling pathway	2.52	**<0.001**	0.55824289	0.331277414	–	–	Neurotrophin signaling is altered in RA (Rihl et al., 2005; Barthel et al., 2009).
hsa04130	SNARE interactions in vesicular transport	0	**<0.001**	0.51353532	0.205302976	0.69465846	0.9727782	
hsa04920	Adipocytokine signaling pathway	2.9	**<0.001**	–	0.999995202	–	–	The adipocytokines and the adipokine network have extensive roles in the pathogenesis of RA (Frommer et al., 2011; Del Prete et al., 2014).
hsa05167	Kaposi sarcoma-associated herpes virus infection	5.91	**<0.001**	–	–	–	–	
hsa04630	JAK-STAT signaling pathway	9.26	**<0.001**	–	0.980050749	–	–	Disruption of the JAK-STAT pathway is a critical event in the pathogenesis and progression of rheumatoid arthritis (Malemud, 2018).
hsa04931	Insulin resistance	1.85	**<0.001**	–	–	–	–	
hsa04260	Cardiac muscle contraction	0	**<0.001**	–	–	0.6976311	1	
hsa05142	Chagas disease (American trypanosomiasis)	7.77	**<0.001**	–	0.999995202	–	–	
hsa05100	Bacterial invasion of epithelial cells	1.35	**<0.001**	–	0.743380146	–	–	Possibly related to generation of neo-autoantigens, molecular mimicry, and bystander activation of the immune system (Li et al., 2013).
hsa05163	Human cytomegalovirus infection	4	**<0.001**	–	–	–	–	
hsa04660	T cell receptor signaling pathway	10.89	**<0.001**	–	0.743380146	–	–	Dysregulation of the TCR signaling pathway was previously implicated in RA biology (Sumitomo et al., 2018).
hsa05131	Shigellosis	3.08	**<0.001**	0.51130292	0.137642182	–	–	Possibly related to generation of neo-autoantigens, molecular mimicry, and bystander activation of the immune system (Li et al., 2013).
hsa05203	Viral carcinogenesis	2.99	**<0.001**	–	0.999995202	–	–	
hsa05166	Human T-cell leukemia virus 1 infection	7.31	**<0.001**	0.48795724	0.137642182	–	–	
hsa04210	Apoptosis	1.47	**<0.001**	–	0.827952041	–	–	Apoptosis may play divergent roles in RA biology (Liu and Pope, 2003).
hsa05165	Human papillomavirus infection	1.82	**<0.001**	–	–	–	–	
hsa05161	Hepatitis B	6.13	**<0.001**	–	–	–	–	
hsa04150	mTOR signaling pathway	0.66	**0.001061744**	–	0.743380146	–	–	Intracellular signaling pathway (including mTOR signaling) play a critical role in rheumatoid arthritis (Malemud, 2013; Malemud, 2015).
hsa05418	Fluid shear stress and atherosclerosis	1.44	**0.001166905**	–	–	–	–	
hsa04218	Cellular senescence	2.5	**0.001351009**	–	–	–	–	
hsa04217	Necroptosis	4.32	**0.001442161**	–	–	–	–	Necroptosis suppresses inflammation via termination of TNF- or LPS-induced cytokine and chemokine production (Kearney et al., 2015).
hsa04145	Phagosome	5.26	**0.001665316**	0.49641734	–	–	–	
hsa03050	Proteasome	2.22	**0.001881322**	–	–	0.7889826	–	Proteasome modulates immune and inflammatory responses in autoimmune diseases (Wang and Maldonado, 2006).
hsa05168	Herpes simplex infection	8.65	**0.002442405**	–	0.53977679	–	–	
hsa05200	Pathways in cancer	4.56	**0.002463658**	–	0.743380146	–	–	
hsa04621	NOD-like receptor signaling pathway	5.06	**0.002477183**	–	0.909381246	–	–	NOD-like receptors are being implicated in the pathology of RA and other rheumatic diseases (McCormack et al., 2009).
hsa05202	Transcriptional misregulation in cancer	4.84	**0.002495122**	–	0.743380146	–	–	
hsa04151	PI3K-Akt signaling pathway	2.82	**0.00331152**	–	–	–	–	PI3K-Akt signaling regulates diverse cellular processes and was proposed as a target for inducing cell death in RA (Malemud, 2015).
hsa05215	Prostate cancer	1.03	**0.003884234**	–	0.999995202	–	–	
hsa05170	Human immunodeficiency virus 1 infection	3.3	**0.004185672**	–	–	–	–	
hsa04066	HIF-1 signaling pathway	5	**0.004382877**	–	–	–	–	Alterations in hypoxia-related signaling pathways are considered potential mechanisms of RA pathogenesis (Quiñonez-Flores et al., 2016).
hsa05225	Hepatocellular carcinoma	3.57	**0.004782642**	–	–	–	–	
hsa04922	Glucagon signaling pathway	0	**0.004927201**	–	–	–	–	
hsa03420	Nucleotide excision repair	0	**0.005418059**	0.63260927	–	–	–	DNA damage load is higher in RA patients, thus activating repair pathways (Lee et al., 2003).
hsa04015	Rap1 signaling pathway	0.97	**0.005543915**	–	–	–	–	Deregulation of Rap1 signaling pathway was shown to be a critical event altering the response of synovial T cells in RA (Remans et al., 2002).
hsa05221	Acute myeloid leukemia	3.03	**0.006008327**	–	0.999995202	–	–	
hsa05132	Salmonella infection	3.49	**0.006557353**	–	0.721697645	–	–	Possibly related to generation of neo-autoantigens, molecular mimicry, and bystander activation of the immune system (Li et al., 2013).
hsa05212	Pancreatic cancer	4	**0.006646458**	–	0.743380146	–	–	
hsa04662	B cell receptor signaling pathway	2.82	**0.00748718**	–	0.851804025	–	–	
hsa04971	Gastric acid secretion	4	**0.008829291**	–	0.743380146	–	–	
hsa04020	Calcium signaling pathway	3.19	**0.009304653**	–	0.999995202	–	–	Dysregulation of the calcium signaling pathway was implicated in RA pathogenesis (Berridge, 2016).
hsa04919	Thyroid hormone signaling pathway	3.45	**0.009478272**	–	–	–	–	
hsa05220	Chronic myeloid leukemia	3.95	**0.011899647**	–	0.743380146	–	–	
hsa04728	Dopaminergic synapse	1.53	**0.012701709**	–	0.743380146	–	–	
hsa05412	Arrhythmogenic right ventricular cardiomyopathy (ARVC)	1.39	**0.012829444**	–	0.96639695	–	–	
hsa04371	Apelin signaling pathway	1.46	**0.015134748**	–	–	–	–	
hsa04910	Insulin signaling pathway	0	**0.015134748**	–	0.999995202	–	0.95215786	
hsa03015	mRNA surveillance pathway	0	**0.015767824**	–	–	–	–	
hsa04658	Th1 and Th2 cell differentiation	17.39	**0.016291789**	–	–	–	–	RA patients were characterized by a disruption of Th1/Th2 balance towards Th1(He et al., 2017).
hsa04620	Toll-like receptor signaling pathway	5.77	**0.017712009**	–	0.743380146	0.72964895	1	Toll-like receptors are being implicated in the pathology of RA and other rheumatic diseases (McCormack et al., 2009).
hsa05410	Hypertrophic cardiomyopathy (HCM)	2.41	**0.019642576**	–	–	–	–	
hsa04668	TNF signaling pathway	5.45	**0.0209396**	–	–	–	–	Intracellular signaling pathway (including TNF signaling) play a critical role in rheumatoid arthritis (Malemud, 2013).
hsa05169	Epstein-Barr virus infection	8.96	**0.022925676**	–	0.743380146	–	–	
hsa05031	Amphetamine addiction	2.94	**0.023901842**	–	0.743380146	–	–	
hsa05414	Dilated cardiomyopathy (DCM)	2.22	**0.025016113**	–	0.851804025	–	–	
hsa04012	ErbB signaling pathway	2.35	**0.026253837**	–	0.999995202	–	–	Intracellular signaling pathway play a critical role in rheumatoid arthritis (Malemud, 2013).
hsa04510	Focal adhesion	0.5	**0.02805129**	–	0.999995202	0.77597433	–	Adhesion molecules have an important role in RA (Pitzalis et al., 1994).
hsa04110	Cell cycle	4.03	**0.029916503**	–	0.743380146	–	–	Cell cycle stalling was recently linked to arthritis (Matsuda et al., 2017).
hsa05206	MicroRNAs in cancer	1.34	**0.03026234**	–	–	–	–	
hsa03460	Fanconi anemia pathway	0	**0.033094195**	–	0.743380146	–	–	DNA damage load is higher in RA patients, thus activating repair pathways (Lee et al., 2003).
hsa05160	Hepatitis C	3.23	**0.035219047**	–	0.743380146	–	–	
hsa04721	Synaptic vesicle cycle	1.28	**0.035442941**	–	–	–	–	
hsa04810	Regulation of actin cytoskeleton	0.47	**0.036496481**	–	0.96639695	0.80830806	–	Actin cytoskeleton dynamics is linked to synovial fibroblast activation (Vasilopoulos et al., 2007). Autoimmune response to cytoskeletal proteins (including actin) was reported in RA (Shrivastav et al., 2002).
hsa04270	Vascular smooth muscle contraction	2.48	**0.036862558**	–	0.070524222	–	1	
hsa05230	Central carbon metabolism in cancer	1.54	**0.038909519**	–	–	–	–	Dysregulation of energy metabolism is indicated in RA (Yang et al., 2015).

“ID” indicates the Kyoto Encyclopedia of Genes and Genomes (KEGG) ID for the enriched pathway, whereas “Pathway” indicates the KEGG pathway name. “% RA genes” indicates the percentage of RA genes in the pathway. The lowest Bonferroni-adjusted p value for pathfindR analysis is provided in “pathfindR,” the false discovery rate (FDR)-adjusted p value for Database for Annotation, Visualization and Integrated Discovery (DAVID) analysis is provided in “DAVID,” the FDR-adjusted p value for Signaling Pathway Impact Analysis (SPIA) is presented in “SPIA,” and the FDR-adjusted p values for Gene Set Enrichment Analysis (GSEA) and GSEAPreranked are presented in“GSEA” and “GSEAPreranked,” respectively. Significant p values (i.e., adjusted p value <0.05) are given in bold font. “-“ indicates the pathway was not found to be enriched by the given tool. If a pathway is relevant to RA, a brief description of its relevance is provided in “Brief Description.”

The summary of results obtained using the different tools and literature support for the identified pathways (where applicable) are presented in **Table 1**. For this dataset, SPIA identified two significant pathways, which were both also identified by pathfindR. No significant pathway was identified by the other tools.

Clustering allowed us to obtain coherent groups of pathways and identify mechanisms relevant to RA, including autoimmune response to the spliceosome (Hassfeld et al., 1995), mechanisms related with response to microbial infection, such as generation of neo-autoantigens and molecular mimicry (Li et al., 2013), dysregulation of various signaling pathways (Remans et al., 2002; Rihl et al., 2005; Barthel et al., 2009; Malemud, 2015), DNA damage repair (Lee et al., 2003), dysregulation of energy metabolism (Yang et al., 2015), and modulation of immune response and inflammation by the proteasome (Wang and Maldonado, 2006).

The activity scores of the representative pathways for each subject indicated that most representative pathways were down-regulated in the majority of subjects (**Figure 2C**).

### The CRC Dataset

**Figure 3:**
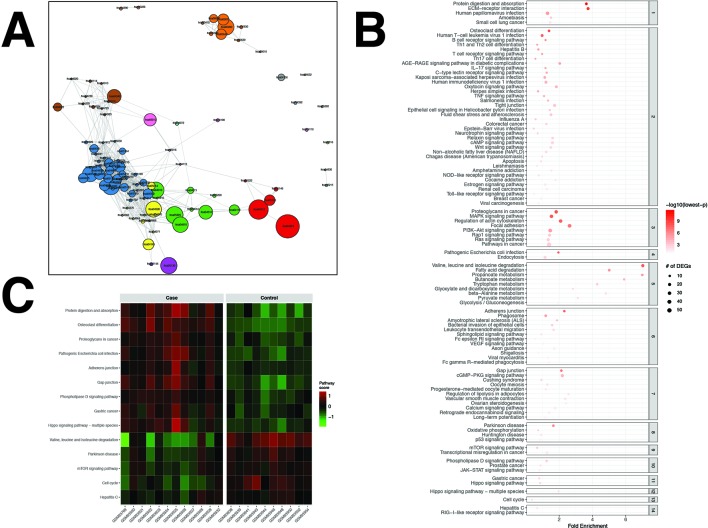
pathfindR enrichment and clustering results on the colorectal cancer (CRC) dataset (lowest p ≤ 0.05). **(A)** Clustering graph, each color displaying the clusters obtained for CRC. Each node is an enriched pathway. The size of a node corresponds to its −log(lowest_p). The thickness of the edges between nodes corresponds to the kappa statistic between the two terms. **(B)** Bubble chart of enrichment results grouped by clusters (labeled on the right-hand side of each panel). The x axis corresponds to fold enrichment values, while the y axis indicates the enriched pathways. The size of the bubble indicates the number of differentially expressed genes (DEGs) in the given pathway. The color indicates the −log10(lowest-p) value; the more it shifts to red, the more significantly the pathway is enriched. **(C)** Heat map of pathway scores per subject. The x axis indicates subjects, whereas the y axis indicates representative pathways. Color scale for the pathway score is provided in the right-hand legend.

For the CRC dataset, 1,356 DEGs were identified (**Supplementary Data Sheet 1**). pathfindR identified 100 significantly enriched pathways (adjusted-p ≤ 0.05) which were partitioned into 14 coherent clusters (**Figures 3A, B**). Forty-eight (48%) of these enriched pathways were relevant to CRC biology, as supported by literature. Brief descriptions of how these are relevant are provided in **Table 2**.

**Table 2:** Pathway analysis results for the colorectal cancer (CRC) dataset (adjusted p < 0.05).

ID	Pathway	% CGC genes	pathfindR	DAVID	SPIA	GSEA	GSEAPreranked	Brief Description
hsa04974	Protein digestion and absorption	5.56	**<0.001**	**0.01573699**	–	–	–	
hsa04512	ECM-receptor interaction	6.1	**<0.001**	**0.00010652**	<0.001	0.3232827	0.92760116	The extracellular matrix modulates the hallmarks of cancer (Pickup et al., 2014).
hsa04380	Osteoclast differentiation	21.26	**<0.001**	–	0.418726575	–	–	
hsa05205	Proteoglycans in cancer	27.86	**<0.001**	**0.02168567**	–	–	–	Proteoglycans play roles in modulating cancer progression, invasion and metastasis (Iozzo and Sanderson, 2011).
hsa05130	Pathogenic Escherichia coli infection	10.91	**<0.001**	0.25769925	**0.015730997**	0.23110063	1	Pathogenic E. coli is claimed to be a cofactor in pathogenesis of colorectal cancer (Bonnet et al., 2014).
hsa00280	Valine, leucine and isoleucine degradation	2.08	**<0.001**	**<0.001**	–	–	–	Degradation of branched chain amino acids could play an important role in the energy supply of cancer cells (Antanaviciute et al., 2017).
hsa04010	MAPK signaling pathway	17.97	**<0.001**	0.08238577	**0.004151739**	0.28760567	1	MAPK signaling plays an important part in progression of colorectal cancer (Fang and Richardson, 2005).
hsa04520	Adherens junction	31.94	**<0.001**	0.0852993	–	0.39334586	0.98078984	Dysregulation of the adherens junction system has particular implications in transformation and tumor invasion (Knights et al., 2012).
hsa04810	Regulation of actin cytoskeleton	15.02	**<0.001**	**0.01469723**	**0.004105905**	0.31124064	1	Regulation of actin cytoskeleton is dysregulated in cancer cell migration and invasion (Yamaguchi and Condeelis, 2007).
hsa05166	Human T-cell leukemia virus 1 infection	27.4	**<0.001**	–	0.858709076	–	–	
hsa04510	Focal adhesion	19.1	**<0.001**	**<0.001**	**<0.001**	0.2348305	0.95611423	Cancer cells exhibit highly altered focal adhesion dynamics (Maziveyi and Alahari, 2017).
hsa04540	Gap junction	19.32	**<0.001**	**0.03853227**	**0.009701705**	0.24327032	0.9830453	Deficiencies in cell-to-cell communication, particularly gap junctional intercellular communication are observed in CRC (Bigelow and Nguyen, 2014).
hsa05012	Parkinson disease	6.34	**<0.001**	**0.28728621**	**0.025978875**	–	0.91026866	
hsa04662	B cell receptor signaling pathway	42.25	**<0.001**	–	0.500093708	0.27041057	1	
hsa00071	Fatty acid degradation	6.82	**<0.001**	**<0.001**	–	–	–	Adipocytes activate mitochondrial fatty acid oxidation and autophagy to promote tumor growth in colon cancer (Wen et al., 2017).
hsa04658	Th1 and Th2 cell differentiation	19.57	**<0.001**	–	–	–	–	T helper cells are important in cancer immunity (Knutson and Disis, 2005).
hsa05165	Human papillomavirus infection	19.09	**<0.001**	–	–	–	–	
hsa05161	Hepatitis B	31.29	**<0.001**	–	–	–	–	
hsa00640	Propanoate metabolism	0	**<0.001**	<0.001	–	–	–	
hsa04151	PI3K-Akt signaling pathway	21.47	**<0.001**	0.07244833	–	–	–	PI3K-Akt signaling is deregulated in CRC (Danielsen et al., 2015; Zhang et al., 2017a).
hsa04660	T cell receptor signaling pathway	31.68	**<0.001**	–	0.698350894	0.44643503	0.965013	T-cell receptor signaling modulates control of anti-cancer immunity (Cronin and Penninger, 2007).
hsa04659	Th17 cell differentiation	26.17	**<0.001**	–	–	–	–	A unique change of Th17 cells was observed in the progression of CRC (Wang et al., 2012).
hsa04933	AGE-RAGE signaling pathway in diabetic complications	31	**<0.001**	–	–	–	–	
hsa04657	IL-17 signaling pathway	9.68	**<0.001**	–	–	–	–	IL-17 is considered as a promoter factor in CRC progression (Wu et al., 2013).
hsa04625	C-type lectin receptor signaling pathway	27.88	**<0.001**	–	–	–	–	C-Type lectin receptors may be targeted for cancer immunity (Yan et al., 2015).
hsa05167	Kaposi sarcoma-associated herpesvirus infection	24.73	**<0.001**	–	–	–	–	
hsa05170	Human immunodeficiency virus 1 infection	16.98	**<0.001**	–	–	–	–	
hsa04921	Oxytocin signaling pathway	13.16	**<0.001**	0.1513106	–	–	–	
hsa05168	Herpes simplex infection	10.81	**<0.001**	–	0.840617856	–	–	
hsa04668	TNF signaling pathway	18.18	**<0.001**	–	–	–	–	TNF-α was shown to promote colon cancer cell migration and invasion (Zhao and Zhang, 2018).
hsa04022	cGMP-PKG signaling pathway	11.04	**<0.001**	0.00352246	–	–	–	cGMP-PKG signaling inhibits cell proliferation and induces apoptosis (Fajardo et al., 2014).
hsa00650	Butanoate metabolism	0	**<0.001**	<0.001	–	–	–	Butanoate has the ability to inhibit carcinogenesis (Goncalves and Martel, 2013).
hsa05132	Salmonella infection	8.14	**<0.001**	–	0.524757851	–	–	
hsa05014	Amyotrophic lateral sclerosis (ALS)	15.69	**<0.001**	0.36800171	0.200174194	0.27973756	1	
hsa04530	Tight junction	11.18	**<0.001**	0.0915822	0.02704172	0.27459267	0.98746127	Dysregulation of tight junctions promote tumorigenesis as well as tumor progression in colorectal cancer (Hollande and Papin, 2013).
hsa04150	mTOR signaling pathway	16.45	**<0.001**	–	0.999999998	0.31433496	1	mTOR signaling is accepted as one of the primary mechanisms for sustaining tumor outgrowth and metastasis and is dysregulated in many cancers, including colorectal cancer (Francipane and Lagasse, 2014).
hsa05120	Epithelial cell signaling in Helicobacter pylori infection	14.71	**<0.001**	–	0.552502996	0.327181	1	
hsa05418	Fluid shear stress and atherosclerosis	18.71	**<0.001**	–	–	–	–	
hsa04015	Rap1 signaling pathway	18.93	**<0.001**	–	–	–	–	Rap1 signaling has roles in tumor cell migration and invasion (Zhang et al., 2017b).
hsa05164	Influenza A	15.79	**<0.001**	–	0.999999998	–	–	
hsa05100	Bacterial invasion of epithelial cells	22.97	**<0.001**	–	0.167771421	–	–	
hsa05146	Amebiasis	12.5	**<0.001**	–	0.418726575	–	–	
hsa00380	Tryptophan metabolism	2.5	**<0.001**	0.0036283	–	–	–	Tryptophan metabolism is a promising target for immunotherapy in CRC (Santhanam et al., 2016).
hsa04072	Phospholipase D signaling pathway	20.55	**0.001079935**	–	–	–	–	Phospholipase D signaling has roles in cell migration, invasion and metastasis (Gomez-Cambronero, 2014).
hsa04014	Ras signaling pathway	18.1	**0.001165472**	–	–	–	–	Ras signaling has roles in colorectal cancer progression, treatment response, prognosis (Zenonos and Kyprianou, 2013).
hsa05210	Colorectal cancer	51.16	**0.00129243**	–	0.177026287	0.5272962	1	The pathway of the disease.
hsa05200	Pathways in cancer	26.81	**0.001394025**	0.01610123	0.004421859	0.23207118	0.99618906	“Meta”-pathway of cancer pathways.
hsa05169	Epstein-Barr virus infection	21.39	**0.001483431**	–	0.999999998	–	–	
hsa04934	Cushing syndrome	22.73	**0.002134647**	–	–	–	–	
hsa00190	Oxidative phosphorylation	3.76	**0.002190056**	–	–	–	1	Glucose metabolism is altered in cancers, including CRC (Fang and Fang, 2016).
hsa04144	Endocytosis	11.48	**0.00223191**	–	–	0.74809563	0.9971049	
hsa04722	Neurotrophin signaling pathway	26.05	**0.00225063**	–	0.869732385	0.22082567	0.9303874	Neurotrophin signaling and related factors were found to clearly exert several biological and clinical features in CRC (Akil et al., 2016).
hsa04926	Relaxin signaling pathway	20.77	**0.002413722**	–	–	–	–	Relaxin signaling has a role in tumor cell growth and differentiation (Silvertown et al., 2003).
hsa04024	cAMP signaling pathway	14.57	**0.002417989**	0.37342574	–	–	–	Dysregulation cAMP signaling was implicated in many cancer types, including CRC (Löffler et al., 2008; Fajardo et al., 2014).
hsa04310	Wnt signaling pathway	17.72	**0.002418113**	–	0.068851256	0.3056234	1	Wnt signaling is a key player in many cancers, responsible for maintenance of cancer stem cells, metastasis and immune control (Zhan et al., 2017).
hsa05226	Gastric cancer	30.87	**0.00267019**	–	–	–	–	
hsa04392	Hippo signaling pathway - multiple species	17.24	**0.002915258**	–	–	–	–	Hippo signaling is involved in the control of intestinal stem cell proliferation and colorectal cancer development (Wierzbicki and Rybarczyk, 2015).
hsa04390	Hippo signaling pathway	16.23	**0.003135632**	–	–	–	–	Hippo signaling is involved in the control of intestinal stem cell proliferation and colorectal cancer development (Wierzbicki and Rybarczyk, 2015).
hsa00630	Glyoxylate and dicarboxylate metabolism	0	**0.003236188**	0.30407411	–	–	–	
hsa04110	Cell cycle	23.39	**0.003442925**	–	0.987280486	–	0.9898676	Dysregulation of the cell cycle is implicated in the biology of many cancers, including CRC (Hartwell and Kastan, 1994; Collins et al., 1997; Jarry et al., 2004).
hsa04932	Non-alcoholic fatty liver disease (NAFLD)	13.42	**0.003808796**	–	–	–	–	
hsa05142	Chagas disease (American trypanosomiasis)	21.36	**0.003899445**	–	0.937326751	–	–	
hsa00410	beta-Alanine metabolism	3.23	**0.005120816**	**0.00196409**	–	–	1	
hsa04670	Leukocyte transendothelial migration	16.07	**0.005646255**	0.35563014	0.167771421	0.27631387	1	
hsa00620	Pyruvate metabolism	5.13	**0.00565919**	0.09639534	–	–	–	Glucose metabolism is altered in cancers, including CRC (Fang and Fang, 2016).
hsa04114	Oocyte meiosis	7.2	**0.006872183**	–	0.792716868	0.72884667	0.97175264	
hsa05215	Prostate cancer	51.55	**0.007778647**	–	0.598712628	0.32108408	1	
hsa04210	Apoptosis	22.06	**0.007963488**	–	0.869732385	–	–	Abnormalities in apoptotic function contribute to both the pathogenesis of colorectal cancer and its resistance to chemotherapeutic drugs and radiotherapy (Watson, 2004).
hsa05140	Leishmaniasis	10.81	**0.008480037**	–	0.999999998	0.24636032	1	
hsa05222	Small cell lung cancer	27.96	**0.008933391**	–	0.120809416	0.45156074	1	
hsa05160	Hepatitis C	22.58	**0.010464676**	–	0.869732385	–	–	
hsa05031	Amphetamine addiction	13.24	**0.010805065**	–	0.107609007	–	–	
hsa04621	NOD-like receptor signaling pathway	6.74	**0.011387668**	–	0.999999998	0.5148187	0.9774175	NOD-like receptors are accepted as master regulators of inflammation and cancer (Saxena and Yeretssian, 2014).
hsa04914	Progesterone-mediated oocyte maturation	16.16	**0.011933047**	–	0.857467386	0.5950144	0.9922697	
hsa04923	Regulation of lipolysis in adipocytes	18.52	**0.011957867**	0.19643837	–	–	–	Adipocytes activate mitochondrial fatty acid oxidation and autophagy to promote tumor growth in colon cancer (Wen et al., 2017).
hsa04071	Sphingolipid signaling pathway	18.64	**0.012088886**	–	–	–	–	Sphingolipids have emerging roles in CRC (García-Barros et al., 2014).
hsa05016	Huntington disease	10.88	**0.013246653**	–	0.494422017	–	1	
hsa05030	Cocaine addiction	16.33	**0.014430369**	–	0.310528247	–	–	
hsa04270	Vascular smooth muscle contraction	12.4	**0.014703261**	**0.01450931**	**<0.001**	0.31157959	0.91536194	
hsa04915	Estrogen signaling pathway	22.06	**0.014973032**	–	–	–	–	
hsa04664	Fc epsilon RI signaling pathway	29.41	**0.016512816**	–	0.552502996	0.7568524	0.99502826	
hsa05211	Renal cell carcinoma	44.93	**0.017251888**	–	0.107609007	0.59724545	1	
hsa05202	Transcriptional misregulation in cancer	44.09	**0.017926078**	–	0.329766057	–	–	Core cancer pathway
hsa04913	Ovarian steroidogenesis	4.08	**0.021805547**	–	–	–	–	
hsa04620	Toll-like receptor signaling pathway	14.42	**0.023494048**	–	0.968714181	0.44691193	1	Toll-like receptor signaling pathway is being considered as a potential therapeutic target in colorectal cancer (Moradi-Marjaneh et al., 2018).
hsa04370	VEGF signaling pathway	33.9	**0.025228423**	–	0.768939947	0.53752804	1	Dysregulation of VEGF signaling is observed in numerous cancers, including CRC (Sun, 2012; Stacker and Achen, 2013).
hsa04020	Calcium signaling pathway	9.57	**0.025565655**	0.33050764	0.057419238	0.3367621	0.9716838	Alterations of calcium signaling modulate tumor initiation, angiogenesis, progression and metastasis (Cui et al., 2017).
hsa05224	Breast cancer	31.29	**0.028494806**	–	–	–	–	
hsa04630	JAK-STAT signaling pathway	24.07	**0.029068433**	–	0.494422017	0.40343955	1	Jak-STAT signaling is involved in immune function and cell growth and has an important role in colorectal cancer (Slattery et al., 2013).
hsa04723	Retrograde endocannabinoid signaling	4.05	**0.029254258**	–	0.147248603	–	–	
hsa04622	RIG-I-like receptor signaling pathway	7.14	**0.030848585**	–	0.524757851	0.95792913	–	RIG-I-like receptors are important in immune signaling (Loo and Gale, 2011).
hsa04720	Long-term potentiation	22.39	**0.031734969**	–	0.899457922	0.7634754	0.9743132	
hsa04360	Axon guidance	14.36	**0.032363714**	0.14566283	**0.03397083**	0.31695387	0.98838806	
hsa04115	p53 signaling pathway	33.33	**0.033554867**	–	0.869732385	–	0.9952885	p53 signaling influences many key processes such as cell cycle arrest, apoptosis, and angiogenesis (Slattery et al., 2018).
hsa05131	Shigellosis	10.77	**0.033710491**	–	0.87420689	–	–	
hsa05203	Viral carcinogenesis	23.38	**0.036540488**	–	0.999999998	–	–	
hsa05416	Viral myocarditis	18.64	**0.038063956**	–	0.418726575	0.27175233	0.9940278	
hsa04666	Fc gamma R-mediated phagocytosis	20.88	**0.039418918**	–	0.141340043	0.32853782	–	
hsa00010	Glycolysis / Gluconeogenesis	1.47	**0.044512411**	0.35158586	–	–	1	Glucose metabolism is altered in cancers, including CRC (Fang and Fang, 2016).
hsa01212	Fatty acid metabolism	0	–	**<0.001**	–	–	1	
hsa01130	Biosynthesis of antibiotics	0	–	**<0.001**	–	–	–	
hsa04924	Renin secretion	7.69	0.050814742	**<0.001**	–	–	–	
hsa05414	Dilated cardiomyopathy	8.89	0.211547395	0.10754894	0.009508921	0.29030624	0.95637035	
hsa03320	PPAR signaling pathway	4.05	–	0.11340534	0.015730997	0.5118186	0.98862046	PPARδ acts as a tumor suppressor in colorectal cancer (You et al., 2015).
hsa01200	Carbon metabolism	0	–	**0.003293423**	–	–	–	
hsa01100	Metabolic pathways	0	–	**0.015541794**	–	–	–	Metabolic reprogramming has consequences at the cellular and molecular level with implications for cancer initiation and growth (Hagland et al., 2013)

“ID” indicates the Kyoto Encyclopedia of Genes and Genomes (KEGG) ID for the enriched pathway, whereas “Pathway” indicates the KEGG pathway name. “% CGC genes” indicates the percentage of Cancer Gene Census (CGC) genes in the pathway. The lowest Bonferroni-adjusted p value for pathfindR analysis is provided in “pathfindR,” the false discovery rate (FDR)-adjusted p value for Database for Annotation, Visualization and Integrated Discovery (DAVID) analysis is provided in “DAVID,” the FDR-adjusted p value for Signaling Pathway Impact Analysis (SPIA) is presented in “SPIA,” and the FDR-adjusted p values for Gene Set Enrichment Analysis (GSEA) and GSEAPreranked are presented in “GSEA” and “GSEAPreranked,” respectively. Significant p values (i.e., adjusted p value < 0.05) are given in bold font. “-“ indicates the pathway was not found to be enriched by the given tool. If a pathway is relevant to CRC, a brief description of its relevance is provided in “Brief Description.”

The results obtained using the different tools and literature support for the identified pathways (where applicable) are presented in **Table 2**. For this dataset, DAVID identified 20 significant pathways, 15 of which were also found by pathfindR (4 out of the remaining 5 were not supported by literature to be relevant to CRC). SPIA identified 13 significantly enriched pathways, 11 of which were also identified by pathfindR. Out of the remaining two enriched pathways, only “PPAR signaling pathway” was related to CRC biology (You et al., 2015). Neither GSEA nor GSEAPreranked yielded any significant pathways for the CRC dataset. The Colorectal cancer pathway was identified to be significantly enriched only by pathfindR.

Upon clustering, 14 clusters were identified (**Figures 3A, B**). These clusters implied processes previously indicated in colorectal cancer, including but not limited to colorectal cancer and related signaling pathways (Fang and Richardson, 2005; Zenonos and Kyprianou, 2013; Francipane and Lagasse, 2014), apoptosis (Watson, 2004), p53 signaling (Slattery et al., 2018), dysregulation of metabolic functions, including glucose metabolism (Fang and Fang, 2016), fatty acid metabolism (Wen et al., 2017), and amino acid metabolism (Santhanam et al., 2016; Antanaviciute et al., 2017), and cell cycle (Hartwell and Kastan, 1994; Collins et al., 1997; Jarry et al., 2004). Brief descriptions of all pathways relevant to CRC are provided in **Table 2**.

Representative pathways that were upregulated in the majority of subjects included important pathways related to cancer in general and colorectal cancer, such as the proteoglycans in cancer, adherens junction, gap junction, and Hippo signaling pathway. Representative pathways that were downregulated in the majority of subjects included other important pathways related to colorectal cancer, such as valine, leucine, and isoleucine degradation, mTOR signaling pathway, and cell cycle (**Figure 3C**).

### The PCa Dataset

**Figure 4:**
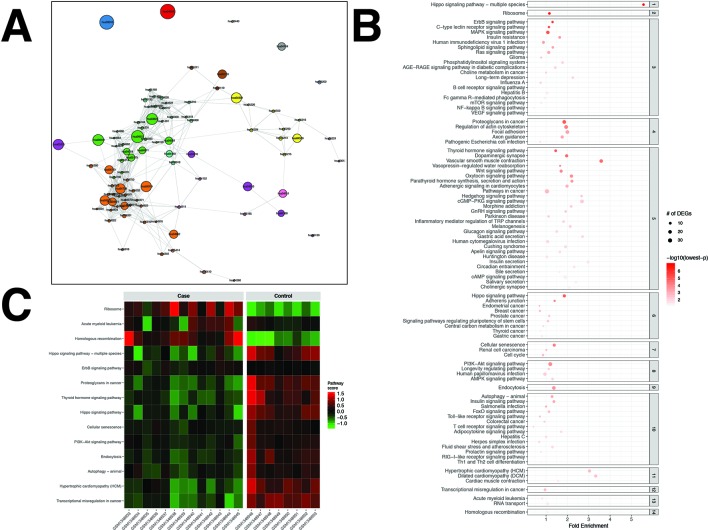
pathfindR enrichment and clustering results on the prostate cancer (PCa) dataset (lowest p ≤ 0.05). **(A)** Clustering graph, each color displaying the clusters obtained for PCa. Each node is an enriched pathway. The size of a node corresponds to its −log(lowest_p). The thickness of the edges between nodes corresponds to the kappa statistic between the two terms. **(B)** Bubble chart of enrichment results grouped by clusters (labeled on the right-hand side of each panel). The x axis corresponds to fold enrichment values, while the y axis indicates the enriched pathways. The size of the bubble indicates the number of differentially expressed genes (DEGs) in the given pathway. The color indicates the −log10(lowest-p) value; the more it shifts to red, the more significantly the pathway is enriched. **(C)** Heat map of pathway scores per subject. The x axis indicates subjects, whereas the y axis indicates representative pathways. Color scale for the pathway score is provided in the right-hand legend.

For the PCa dataset, 1,240 DEGs were identified (**Supplementary Data Sheet 1**). pathfindR identified 92 significantly enriched pathways (adjusted-p ≤ 0.05) which were clustered into 14 coherent clusters (**Figures 4A, B**). Forty-six (50%) of these enriched pathways were relevant to PCa biology, as supported by literature. Brief descriptions of the relevancies are provided in **Table 3**.

**Table 3:** Pathway analysis results for the prostate cancer (PCa) dataset (adjusted p < 0.05).

ID	Pathway	% CGC genes	pathfindR	DAVID	SPIA	GSEA	GSEAPreranked	Brief Description
hsa04392	Hippo signaling pathway - multiple species	17.24	**<0.001**	–	–	–	–	The hippo pathway effector YAP regulates motility, invasion, and castration-resistant growth of prostate cancer cells (Zhang et al., 2015).
hsa03010	Ribosome	1.96	**<0.001**	–	–	0.425191	1	Certain ribosomal proteins are altered and may serve as putative biomarkers for prostate cancer (Arthurs et al., 2017).
hsa04012	ErbB signaling pathway	40	**<0.001**	–	0.637063484	–	–	There are interactions among the ErbB receptor network, its downstream pathways, and androgen receptor signaling (El Sheikh et al., 2003).
hsa04625	C-type lectin receptor signaling pathway	27.88	**<0.001**	–	–	–	–	C-type lectin receptors are emerging orchestrators of sterile inflammation and represent potential therapeutic targets in many cancers, including PCa (Chiffoleau, 2018). C-type lectins were shown to facilitate tumor metastasis (Ding et al., 2017).
hsa04010	MAPK signaling pathway	17.97	**<0.001**	–	0.282733912	–	0.90003437	MAPK signaling pathways act through their effects on apoptosis, survival, metastatic potential, and androgen-independent growth in prostate cancer (Rodríguez-Berriguete et al., 2012).
hsa05205	Proteoglycans in cancer	27.86	**<0.001**	**0.02399376**	–	–	–	Proteoglycans play roles in modulating cancer progression, invasion and metastasis (Iozzo and Sanderson, 2011).
hsa04919	Thyroid hormone signaling pathway	32.76	**<0.001**	0.5109672	–	–	–	
hsa04390	Hippo signaling pathway	16.23	**<0.001**	0.10679672	–	–	–	The hippo pathway effector YAP regulates motility, invasion, and castration-resistant growth of prostate cancer cells (Zhang et al., 2015).
hsa04728	Dopaminergic synapse	11.45	**<0.001**	0.06897641	**0.034643839**	–	–	
hsa04270	Vascular smooth muscle contraction	12.4	**<0.001**	**<0.001**	**<0.001**	–	0.9954409	
hsa04810	Regulation of actin cytoskeleton	15.02	**<0.001**	**0.02727598**	**0.033699531**	–	–	Dysregulated in cancer cell migration and invasion (Yamaguchi and Condeelis, 2007).
hsa04218	Cellular senescence	25.63	**<0.001**	–	–	–	–	Cellular senescence may play a role in treatment resistance in PCa (Blute et al., 2017).
hsa04520	Adherens junction	31.94	**<0.001**	–	–	–	–	Dysregulation of the adherens junction system has particular implications in transformation and tumor invasion (Knights et al., 2012).
hsa04962	Vasopressin-regulated water reabsorption	13.64	**<0.001**	–	0.655412336	–	–	
hsa04310	Wnt signaling pathway	17.72	**<0.001**	0.14714863	0.174150166	–	–	Wnt signaling is implicated in PCa biology (Murillo-Garzon and Kypta, 2017).
hsa04151	PI3K-Akt signaling pathway	21.47	**<0.001**	–	–	–	–	Activation of PI3K-Akt signaling pathway promotes prostate cancer cell invasion (Shukla et al., 2007).
hsa04921	Oxytocin signaling pathway	13.16	**<0.001**	0.09939094	–	–	–	Oxytocin signaling has a role in prostate cancer metastasis (Zhong et al., 2010).
hsa04144	Endocytosis	11.48	**<0.001**	0.14183304	–	–	–	Defective vesicular trafficking of growth factor receptors, as well as unbalanced recycling of integrin- and cadherin-based adhesion complexes, has emerged as a multifaceted hallmark of malignant cells (Mosesson et al., 2008).
hsa04928	Parathyroid hormone synthesis, secretion and action	20.75	**<0.001**	–	–	–	–	
hsa04931	Insulin resistance	14.81	**<0.001**	0.44248049	–	–	–	Men in the highest tertile of insulin resistance (IR) had an increased risk of prostate cancer, indicating a potential pathogenetic link of IR with prostate cancer (Hsing et al., 2003).
hsa05170	Human immunodeficiency virus 1 infection	16.98	**<0.001**	–	–	–	–	
hsa04071	Sphingolipid signaling pathway	18.64	**<0.001**	–	–	–	–	Sphingolipids are modulators of cancer cell death and represent potential therapeutic targets (Segui et al., 2006; Shaw et al., 2018).
hsa04510	Focal adhesion	19.1	**<0.001**	**0.01182864**	**0.003797795**	–	–	Cancer cells exhibit highly altered focal adhesion dynamics (Maziveyi and Alahari, 2017).
hsa04014	Ras signaling pathway	18.1	**<0.001**	–	–	–	–	Ras signaling plays an important role in prostate cancer progression and is a possibly mediator of hormone resistance (Weber and Gioeli, 2004; Whitaker and Neal, 2010).
hsa04140	Autophagy - animal	17.19	**<0.001**	–	0.91466497	0.7367432	–	Autophagy is a modulator of PCa biology and is a therapeutic target (Farrow et al., 2014).
hsa04360	Axon guidance	14.36	**<0.001**	0.36615434	0.174150166	–	–	
hsa04910	Insulin signaling pathway	18.98	**<0.001**	–	0.592610905	–	–	Insulin signaling has crucial roles in cell proliferation and death. Insulin receptors were detected on primary human prostate cancers (Cox et al., 2009; Bertuzzi et al., 2016).
hsa05132	Salmonella infection	8.14	**0.001024926**	–	0.884388639	–	–	
hsa04261	Adrenergic signaling in cardiomyocytes	11.81	**0.001251519**	0.1359051	–	–	–	
hsa05213	Endometrial cancer	60.34	**0.001571998**	–	0.889535144	0.9776995	0.9350631	
hsa05211	Renal cell carcinoma	44.93	**0.001704596**	–	0.958690885	–	–	
hsa05200	Pathways in cancer	26.81	**0.001864931**	0.44205232	0.592610905	–	–	“Meta”-pathway of cancer pathways.
hsa05214	Glioma	44	**0.00191144**	–	0.678606672	–	–	
hsa04110	Cell cycle	23.39	**0.00200072**	–	0.53576482	0.73860705	–	Dysregulation of the cell cycle is implicated in the biology of many cancers, including PCa (Hartwell and Kastan, 1994; Collins et al., 1997; Balk and Knudsen, 2008).
hsa05410	Hypertrophic cardiomyopathy (HCM)	6.02	**0.002088682**	**0.01508581**	–	–	0.94539815	
hsa05202	Transcriptional misregulation in cancer	44.09	**0.002227785**	–	0.909985754	–	–	Core cancer pathway.
hsa04068	FoxO signaling pathway	29.55	**0.00256445**	–	–	–	–	FOXO signaling is implicated and considered as a therapeutic target in many cancers, including PCa (Farhan et al., 2017).
hsa04620	Toll-like receptor signaling pathway	14.42	**0.002757387**	–	0.999737262	1	–	TLRs may serve as a double-edged sword in prostate cancer tumorigenesis by promoting malignant transformation of epithelial cells and tumor growth, or on the contrary, inducing apoptosis, and inhibiting tumor progression (Zhao et al., 2014).
hsa05414	Dilated cardiomyopathy (DCM)	8.89	**0.002884316**	**0.00445895**	**0.00145624**	–	0.9310661	
hsa05224	Breast cancer	31.29	**0.002996465**	–	–	–	–	
hsa04340	Hedgehog signaling pathway	21.28	**0.003701704**	–	0.603911642	–	–	Hedgehog signaling plays an important role in the development and progression of PCa (Gonnissen et al., 2013).
hsa05215	Prostate cancer	51.55	**0.004678127**	–	0.637063484	–	–	The pathway of the disease.
hsa04211	Longevity regulating pathway	25.84	**0.004704898**	–	–	–	–	
hsa04022	cGMP-PKG signaling pathway	11.04	**0.004947514**	**0.00142051**	–	–	–	cGMP-PKG signaling inhibits cell proliferation and induces apoptosis (Fajardo et al., 2014).
hsa05032	Morphine addiction	4.4	**0.005138281**	0.37736294	0.174150166	–	–	
hsa04550	Signaling pathways regulating pluripotency of stem cells	31.65	**0.00540706**	–	–	–	–	
hsa04912	GnRH signaling pathway	19.35	**0.005600378**	0.37736294	0.340227111	–	0.9284794	GnRH signaling has roles in cancer cell proliferation and metastasis in many cancers, including PCa (Gründker and Emons, 2017).
hsa05165	Human papillomavirus infection	19.09	**0.005787239**	–	–	–	–	HPV infection is associated with increasing risk of PCa, indicating a potential pathogenetic link between HPV and prostate cancer (Yin et al., 2017).
hsa05012	Parkinson disease	6.34	**0.005882045**	–	0.895565575	–	–	
hsa04070	Phosphatidylinositol signaling system	6.06	**0.007170858**	0.47255633	0.592215095	–	–	Deregulation PI3 kinase signaling is implicated in prostate carcinogenesis (Elfiky and Jiang, 2013).
hsa04750	Inflammatory mediator regulation of TRP channels	10.1	**0.007170858**	0.47255633	–	–	–	TRP channels have emerged as key proteins in central mechanisms of the carcinogenesis such as cell proliferation, apoptosis and migration (Gkika and Prevarskaya, 2011).
hsa04933	AGE-RAGE signaling pathway in diabetic complications	31	**0.007170858**	–	–	–	–	
hsa05231	Choline metabolism in cancer	27.27	**0.007170858**	–	–	–	–	Core cancer pathway. Choline metabolites can be used as potential prognostic biomarkers for the management of prostate cancer patients (Awwad et al., 2012).
hsa04730	Long-term depression	23.33	**0.007411265**	0.29433246	0.228920589	–	–	
hsa04152	AMPK signaling pathway	15.83	**0.007412407**	–	–	–	–	First identified as a master regulator of metabolism, AMPK may have numerous roles beyond metabolism. AMPK signaling can have context-dependent effects in prostate cancer (Khan and Frigo, 2017).
hsa05210	Colorectal cancer	51.16	0.007572536	–	0.53576482	–	0.8859366	
hsa04660	T cell receptor signaling pathway	31.68	0.007759806	–	0.999737262	–	–	T-cell receptor signaling modulates control of anti-cancer immunity (Cronin and Penninger, 2007).
hsa04916	Melanogenesis	20.79	0.007759806	0.23117007	0.191012563	–	–	
hsa04922	Glucagon signaling pathway	11.65	**0.008383558**	–	–	–	–	
hsa04971	Gastric acid secretion	8	**0.008625239**	0.17201492	0.174150166	–	–	
hsa05164	Influenza A	15.79	**0.009418672**	–	0.871606688	–	–	
hsa05230	Central carbon metabolism in cancer	49.23	**0.00943342**	–	–	–	–	Core cancer pathway.
hsa05163	Human cytomegalovirus infection	24	**0.010897057**	–	–	–	–	
hsa04920	Adipocytokine signaling pathway	18.84	**0.011289828**	–	0.573213367	–	–	Adipocytokines are implicated in many cancers, including PCa (Housa et al., 2006).
hsa05130	Pathogenic Escherichia coli infection	10.91	**0.012638019**	–	0.914414969	–	–	
hsa05160	Hepatitis C	22.58	**0.012701709**	–	0.952731561	–	–	
hsa05168	Herpes simplex infection	10.81	**0.012818879**	–	0.999737262	–	–	
hsa04934	Cushing syndrome	22.73	**0.012968007**	–	–	–	–	
hsa04662	B cell receptor signaling pathway	42.25	**0.015323796**	–	0.871606688	–	–	
hsa05418	Fluid shear stress and atherosclerosis	18.71	**0.016016719**	–	–	–	–	
hsa05216	Thyroid cancer	70.27	**0.016672034**	–	0.77019655	–	–	
hsa05221	Acute myeloid leukemia	50	**0.018273818**	–	0.916809245	0.9737256	0.9253648	
hsa04371	Apelin signaling pathway	13.14	**0.019388476**	–	–	–	–	Various apelin peptides can stimulate tumor growth and proliferation of many types of cancer cells, including PCa (Wysocka et al., 2018).
hsa05016	Huntington disease	10.88	**0.019575207**	–	0.887106943	1	0.9790702	
hsa04911	Insulin secretion	11.76	**0.021091491**	**0.02334547**	–	–	–	
hsa04917	Prolactin signaling pathway	31.43	**0.021797194**	–	–	–	–	Prolactin signalling promotes prostate tumorigenesis and may be targeted for therapy (Goffin et al., 2011; Sackmann-Sala and Goffin, 2015).
hsa03440	Homologous recombination	24.39	**0.027879334**	–	–	0.82518643	0.88681024	Homologous recombination offers a model for novel DNA repair targets and therapies in PCa (Bristow et al., 2007).
hsa04713	Circadian entrainment	10.31	**0.028299959**	0.11376372	–	–	–	
hsa03013	RNA transport	5.45	**0.029590861**	–	0.887106943	–	–	Many common and specialized mRNA export factors are dysregulated in cancer (Siddiqui and Borden, 2012).
hsa04260	Cardiac muscle contraction	6.41	**0.030119546**	–	–	–	0.92371947	
hsa05161	Hepatitis B	31.29	**0.031074222**	–	–	–	–	
hsa04666	Fc gamma R-mediated phagocytosis	20.88	**0.032172546**	–	0.838868067	–	–	
hsa04976	Bile secretion	4.23	**0.032183139**	–	0.77019655	–	–	
hsa04024	cAMP signaling pathway	14.57	**0.035154932**	0.12151133	–	–	–	Dysregulation cAMP signaling was implicated in many cancer types, including PCa (Fajardo et al., 2014).
hsa05226	Gastric cancer	30.87	**0.035466235**	–	–	–	–	
hsa04622	RIG-I-like receptor signaling pathway	7.14	**0.036168176**	–	0.678606672	0.998692	1	
hsa04150	mTOR signaling pathway	16.45	**0.03639603**	–	0.608898009	0.97279966	0.8907857	mTOR signaling is implicated in prostate cancer progression and androgen deprivation therapy resistance (Edlind and Hsieh, 2014).
hsa04064	NF-kappa B signaling pathway	17.89	**0.036565869**	–	0.999737262	–	–	The NF-kappa B signaling pathway controls the progression of Pca (Jin et al., 2008).
hsa04970	Salivary secretion	6.67	**0.038144831**	0.35044831	0.228920589	–	–	
hsa04658	Th1 and Th2 cell differentiation	19.57	**0.040720473**	–	–	–	–	T helper cells are important in cancer immunity (Knutson and Disis, 2005).
hsa04370	VEGF signaling pathway	33.9	**0.043130708**	–	0.889535144	–	–	Angiogenesis has been shown to play an important role in tumorigenesis, proliferation and metastasis in PCa. Various promising agents that target VEGF signaling have been tested (Aragon-Ching and Dahut, 2009).
hsa04725	Cholinergic synapse	18.75	**0.04793374**	0.13876451	0.129551973	–	–	
hsa00120	Primary bile acid biosynthesis	0	–	–	–	0.78211117	**<0.001**	

“ID” indicates the Kyoto Encyclopedia of Genes and Genomes (KEGG) ID for the enriched pathway, whereas “Pathway” indicates the KEGG pathway name. “% CGC genes” indicates the percentage of Cancer Gene Census (CGC) genes in the pathway. The lowest Bonferroni-adjusted p value for pathfindR analysis is provided in “pathfindR,” the false discovery rate (FDR)-adjusted p value for Database for Annotation, Visualization and Integrated Discovery (DAVID) analysis is provided in “DAVID,” the FDR-adjusted p value for Signaling Pathway Impact Analysis (SPIA) is presented in “SPIA,” and the FDR-adjusted p values for Gene Set Enrichment Analysis (GSEA) and GSEAPreranked are presented in “GSEA” and “GSEAPreranked,” respectively. Significant p values (i.e., adjusted p value < 0.05) are given in bold font. “-“ indicates the pathway was not found to be enriched by the given tool. If a pathway is relevant to PCa, a brief description of its relevance is provided in “Brief Description.”

The results obtained using the different tools and literature support for the identified pathways (where applicable) are presented in **Table 3**. DAVID identified eight significant pathways, which were all also identified by pathfindR and only half of which were relevant to PCa. SPIA identified five significantly enriched pathways, all of which were also identified by pathfindR. GSEA identified no significant pathways, whereas GSEAPreranked identified one significant pathway, for which no association with PCa was provided by the literature. The prostate cancer pathway was identified to be significantly enriched only by pathfindR.

The clusters identified by pathfindR pointed to several mechanisms previously shown to be important for prostate cancer. These mechanisms included but were not limited to the prostate cancer pathway and related signaling pathways (El Sheikh et al., 2003; Shukla et al., 2007; Rodríguez-Berriguete et al., 2012), cancer immunity (Knutson and Disis, 2005; Zhao et al., 2014), Hippo signaling (Zhang et al., 2015), cell cycle (Balk and Knudsen, 2008), autophagy (Farrow et al., 2014), and insulin signaling (Cox et al., 2009; Bertuzzi et al., 2016).

The majority of representative pathways relevant to PCa were down-regulated (**Figure 4C**).

### Common Pathways Between the CRC and PCa Datasets

Because the CRC and PCa datasets were both cancers, they were expected to have common pathways identified by pathfindR. Indeed, 47 common significant pathways (adjusted-p ≤ 0.05) were identified (**Supplementary Table 1**). These common pathways included general cancer-related pathways, such as pathways in cancer, proteoglycans in cancer, MAPK signaling pathway, Ras signaling pathway, Hippo signaling pathway, mTOR signaling pathway, Toll-like receptor signaling pathway, Wnt signaling pathway, and adherens junction.

### Disease-Related Genes in the Significantly Enriched Pathways

The percentages of disease-related genes for each pathway found to be enriched by any tool (adjusted-p ≤ 0.05) are presented in the corresponding columns of **Tables 1**, **2**, and **3** (“% RA Genes” for the RA dataset and “% CGC Genes” for the CRC and PCa datasets). These percentages show great variability but support the literature search results in assessing the disease-relatedness of the enriched pathways.

**Figure 5:**
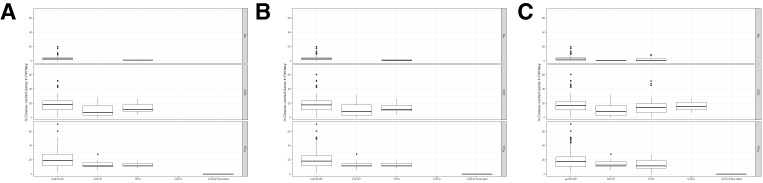
Distributions of disease-associated genes in the enriched pathways. Boxplots displaying the distributions of the percentages of disease-related genes in the pathways found to be enriched by pathfindR, Database for Annotation, Visualization and Integrated Discovery (DAVID), Signaling Pathway Impact Analysis (SPIA), Gene Set Enrichment Analysis (GSEA), and GSEAPreranked in the datasets rheumatoid arthritis (RA), colorectal cancer (CRC), and prostate cancer (PCa). No boxplot for a tool in a particular dataset indicates that the given tool did not identify any enriched pathways in the given dataset. **(A)** Boxplots for all the results filtered for adjusted-p ≤ 0.05. **(B)** Boxplots for all the results filtered for adjusted-p ≤ 0.1. **(C)** Boxplots for all the results filtered for adjusted-p ≤ 0.25.

The distributions of disease-related gene percentages in pathways identified by each tool in the three different datasets, filtered by the adjusted-p value thresholds of 0.05, 0.1, and 0.25, are presented in **Figure 5**. As stated before, for the RA dataset, only pathfindR and SPIA identified significant pathways. The median percentages of RA-associated genes of the enriched pathways of pathfindR was higher than the median percentages of SPIA (2.43% vs. 0.96% for the 0.05 cutoff, 2.5% vs. 0.61% for the 0.1 cutoff, and 2.27% vs. 0.67% for the 0.25 cutoff). For CRC, pathfindR displayed the highest median percentage of CGC genes for all the cutoff values (17.84%, 17.72%, and 16.7% for 0.05, 0.1, and 0.25, respectively). For the PCa dataset, the median percentages of CGC genes of the enriched pathways of pathfindR were again the highest among all tools for all significance cutoff values (18.73%, 18.37%, and 17.93% for 0.05, 0.1, and 0.25, respectively).

### Permutation Assessment

**Figure 6:**
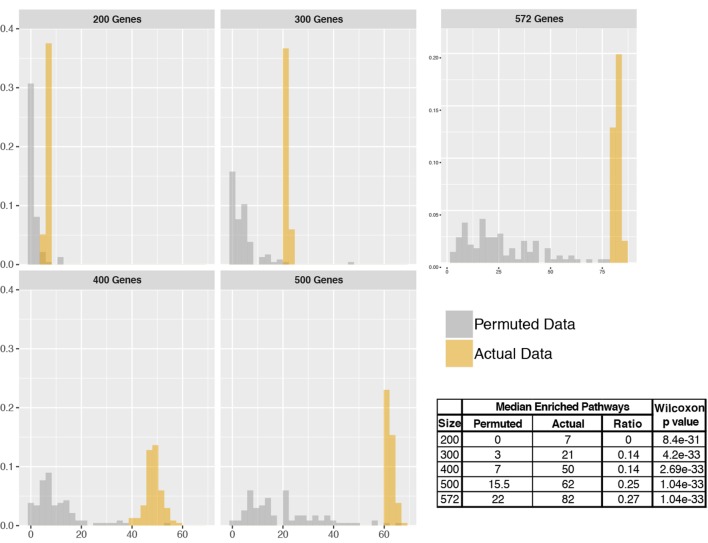
Distributions of the number of enriched pathways for actual vs. permuted data. Histograms displaying the distributions of the number of enriched pathways for actual and permuted data for input sizes of 200, 300, 400, 500, and 572. The x axes correspond to the number of enriched pathways, and the y axes correspond to relative frequencies. On the right bottom, a table summarizing the results is provided.

To assess the number of pathways identified to be enriched by pathfindR, we performed analyses using actual and permuted data of different sizes. Comparison of the distributions of actual vs. permuted data is presented in **Figure 6**. Wilcoxon rank sum tests revealed that the distributions of the numbers of enriched pathways obtained using actual and permuted input data were significantly different (all p < 0.001). The median number of enriched pathways was lower for permuted data in each case.

It was observed that the ratio of the median number of pathways (permuted/actual) tended to increase as the number of input genes increased. This is most likely because as the input size gets larger, there is higher chance in finding highly connected subnetworks that in turn leads to identifying a higher number of enriched pathways.

### Assessment of the Effect of DEGs Without Any Interactions on Enrichment Results

To gain further support for our proposal that directly performing enrichment analysis on a list of genes is not completely informative because this ignores the interaction information, we performed ORA (as implemented in pathfindR) on (i) all of the DEG lists (RA, CRC, and PCa) and (ii) the filtered list of DEGs for the same datasets so that they only contain DEGs found in the Biogrid PIN. This allowed us to assess any effect of eliminating DEGs with no interactions on the enrichment results.

The numbers of DEGs found in the Biogrid PIN for each dataset was as follows: RA—481 (out of 572 total), CRC—989 (out of 1,356) and PCa—900 (out of 1,240). The ORA results are presented in **Supplementary Data Sheet 3**. The elimination of DEGs without any interaction clearly affected numbers of significantly enriched (FDR < 0.05) KEGG pathways (**Supplementary Table 2**). For the RA dataset, no significantly enriched pathways were found using all DEGs, whereas elimination of non-interacting DEGs resulted in one significant pathway. For CRC and PCa, using only DEGs found in the PIN, the number of significantly enriched pathways were doubled compared to using all of the genes without taking into account any interaction information. We would like to note that these results partly explain why taking interaction information into account results in enhanced enrichment results.

### Assessment of the Effect of PINs on Enrichment Results

To assess any effect of the choice of PIN on pathfindR results, we first compared the default PINs in terms of the interactions they contain. The number of interactions in the PINs were as follows: 289,417 interactions in Biogrid, 79,741 interactions in GeneMania, 121,007 interactions in IntAct, and 53,047 interactions in KEGG. The numbers of common interactions between any pair of PINs and the overlap percentages of the interactions are presented in **Supplementary Table 3**. The results show that there is very little overlap between the PINs. Despite the fact that Biogrid has more than double the interactions of IntAct and 3 times the interactions of GeneMania, it remarkably does not contain half of the interactions they contain, implying this lack of overlap between PINs may affect pathfindR results.

We then proceeded with analyzing any effect of the choice of PIN on active-subnetwork-oriented pathway enrichment analysis. Venn diagrams comparing enrichment results obtained through pathfindR analyses with all available PINs are presented in **Supplementary Figure 1**. This comparison revealed that there was no compelling overlap among the enriched pathways obtained by using different PINs. Overall, using Biogrid and KEGG resulted in the highest number of significantly enriched pathways for all datasets.

As described in Materials and Methods, the results presented in this subsection were obtained using greedy search with search depth of 1 and maximum depth of 1, which results in multiple subnetworks structured as local subnetworks. Although it is not fully dependent on it, this method requires direct interactions between input genes. In the extreme case where there is no direct connection between any pair of two input genes, it is impossible to get any multi-node subnetworks with this method. Therefore, in order to gain a better understanding of the lack of overlap between the enrichment results presented above, we analyzed the numbers of direct interactions of input genes in each PIN. These results are presented as Venn diagrams in **Supplementary Figure 2**. It is striking that there are only nine common interactions of RA DEGs in all PINs (although there are 54 common interactions in PINs except KEGG). The findings are similar for the CRC and PCa datasets: there are 11 common CRC DEG interactions in all PINs (81 in PINs except KEGG), and 5 PCa DEG interactions (56 in PINs except KEGG).

In case of utilizing KEGG PIN and KEGG pathways, the same interactions for both subnetwork interaction and enrichment analysis are considered. This approach does not introduce any extra information to the analysis, and it is clear that interacting gene groups in the KEGG PIN will be enriched in KEGG pathways. This explains the high number of pathways obtained using the KEGG PIN. Moreover, it is known that pathways in pathway databases may be strongly biased by some classes of genes or phenotypes that are popular targets, such as cancer signaling (Liu et al., 2017a). Therefore, the PIN obtained through KEGG pathway interactions are biased. Biogrid has the highest coverage for direct interactions among DEGs as seen in **Supplementary Figure 2**. It is unbiased in terms of phenotypes, and using Biogrid to extract KEGG pathways combines the two sources of information. 

Considering all of the above-mentioned findings, we conclude that utilizing the Biogrid PIN can provide the researcher with the most extensive enrichment results.

## Discussion

PathfindR is an R package that enables active subnetwork-oriented pathway analysis, complementing the gene-phenotype associations identified through differential expression/methylation analysis. 

In most gene set enrichment approaches, relational information captured in the graph structure of a PIN is overlooked. Hence, during these analyses, genes in the network neighborhood of significant genes are not taken into account. The approach we considered for exploiting interaction information to enhance pathway enrichment analysis was active subnetwork search. In a nutshell, active subnetwork search enables inclusion of genes that are not significant genes themselves but connect significant genes. This results in the identification of phenotype-associated connected significant subnetworks. Initially identifying active subnetworks in a list of significant genes and then performing pathway enrichment analysis of these active subnetworks efficiently exploits interaction information between the genes. This, in turn, helps uncover relevant phenotype-related mechanisms underlying the disease, as demonstrated in the example applications.

Through pathfindR, numerous relevant pathways were identified in each example. The literature-supported disease-related pathways mostly ranked higher in the pathfindR results. The majority of additional pathways identified through pathfindR were relevant to the pathogenesis of the diseases under study, as supported by literature. A separate confirmation of disease-relatedness was provided by analysis of the distributions of the percentage of disease genes in the identified pathways. This analysis revealed that pathfindR pathways contained the highest median percentages of disease-related genes in each dataset regardless of significance cutoff value, implying that the pathways identified by pathfindR are indeed associated with the given disease. Together, these two assessments of disease-relatedness of pathways indicate that pathfindR produces pathway enrichment results at least as relevant as the other tools widely used for enrichment analysis.

We propose that pathfindR performed better than the analyzed pathway analysis tools because, for enrichment analysis, it included disease-related genes that were not in the DEG list but that were known to interact with the DEGs, which most enrichment tools disregard. By performing enrichment analyses on distinct sets of interacting genes (i.e., active subnetworks), pathfindR also eliminated “false positive” genes that lacked any strong interaction. The above findings indicate that incorporating interaction information prior to enrichment analysis results in better identification of disease-related mechanisms.

This package extends the use of the active-subnetwork-oriented pathway analysis approach to omics data. Additionally, it provides numerous improvements and useful new features. The package provides three active subnetwork search algorithms. The researcher is therefore able to choose between the different algorithms to obtain the optimal results. For the greedy and simulated annealing active subnetwork search algorithms, the search and enrichment processes are executed several times. By summarizing results over the iterations and identifying consistently enriched pathways, the stochasticity of these algorithms is overcome. Additionally, the researcher is able to choose from several built-in PINs and can use their own custom PIN by providing the path to the SIF file. The researcher is also able to choose from numerous built-in gene sets, listed above, and can also provide a custom gene set resource. pathfindR also allows for clustering of related pathways. This allows for combining relevant pathways together, uncovering coherent “meta-pathways” and reducing complexity for easier interpretation of findings. This clustering functionality also aids in eliminating falsely enriched pathways that are initially found because of their similarity to the actual pathway of interest. The package also allows for scoring of pathways in individual subjects, denoting the pathway activity. Finally, pathfindR is built as a stand-alone package, but it can easily be integrated with other tools, such as differential expression/methylation analysis tools, for building fully automated pipelines.

To the best of our knowledge, pathfindR is the first and, so far, the only R package for active-subnetwork-oriented pathway enrichment analysis. It also offers functionality for pathway clustering, scoring, and visualization. All features in pathfindR work together to enable identification and further investigation of dysregulated pathways that potentially reflect the underlying pathological mechanisms. We hope that this approach will allow researchers to better answer their research questions and discover mechanisms underlying the phenotype being studied.

## Author Contributions

OS, OO, and EU conceived the pathway analysis approach. OO and EU implemented the R package. EU performed the analyses presented in this article. OUS, OO, and EU interpreted the results. All authors were involved in the writing of the manuscript and read and approved the version being submitted.

## Conflict of Interest Statement

The authors declare that the research was conducted in the absence of any commercial or financial relationships that could be construed as a potential conflict of interest.

## Supplementary Material

Supplementary Figure 1 Venn diagram of enrichment results obtained through pathfindR analyses with all available PINs.

Supplementary Figure 2 Venn diagram of the numbers of direct interactions of input genes in each PIN.

Supplementary Datasheet 1 The results of differential expression analyses for RA, CRC and PCa, prior to filtering (differential expression statistics for all probes) and after filtering (lists of DEGs).

Supplementary Datasheet 2 The unfiltered results of enrichment analyses using all the different methods on each of the datasets.

Supplementary Datasheet 3 ORA results using all DEGs and using only DEGs found in the BioGRID PIN for each dataset.
