# Supplementary figures and images for: pathfindR: An R Package for Comprehensive Identification of Enriched Pathways in Omics Data Through Active Subnetworks
Source: Front Genet. 2019 Sep 25;10:858. doi: 10.3389/fgene.2019.00858 (PMC6773876; doi:10.3389/fgene.2019.00858)

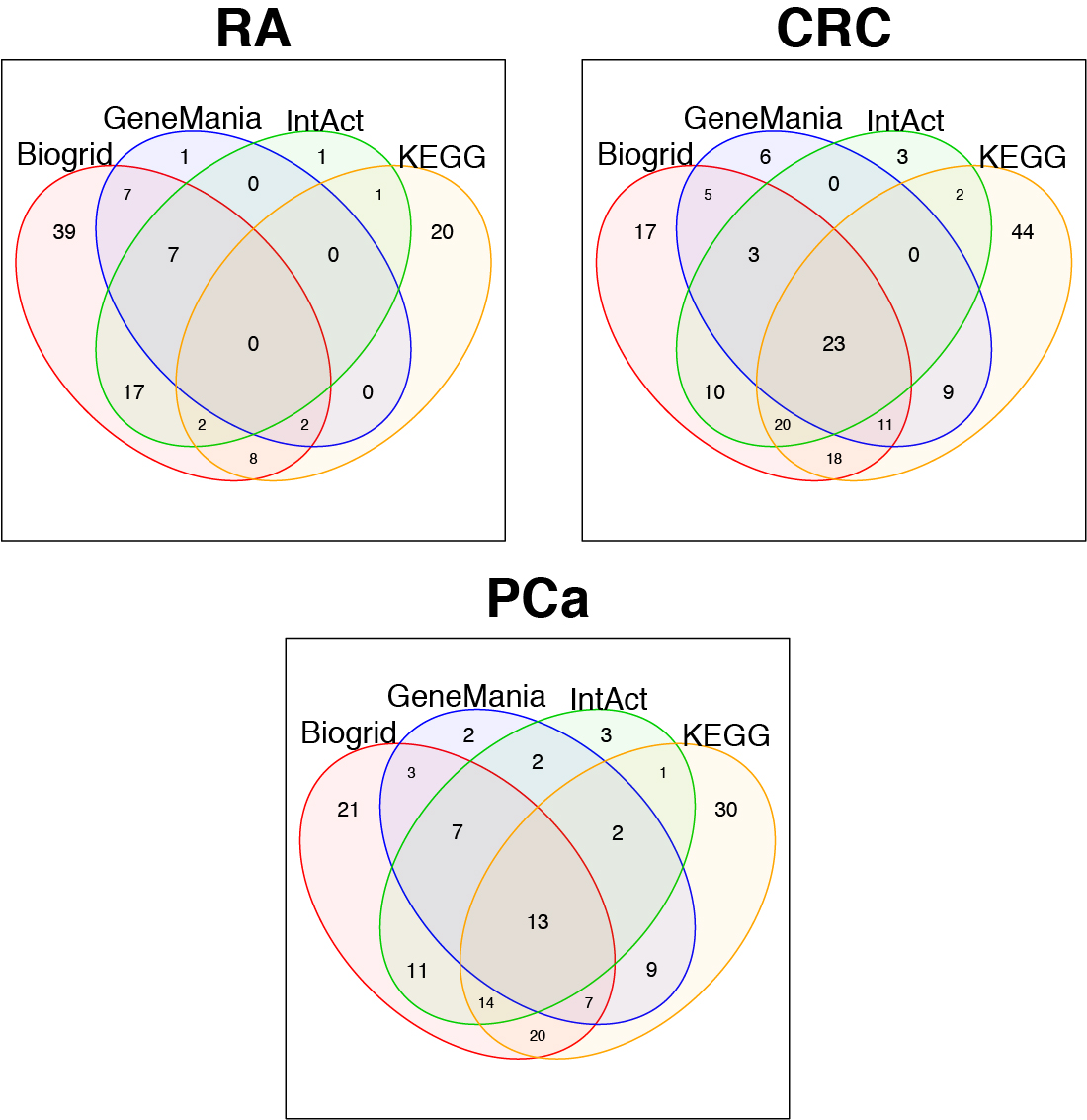

Supplement: Supplementary Figure 1 — Venn diagram of enrichment results obtained through pathfindR analyses with all available PINs. [file Image_1.jpeg]

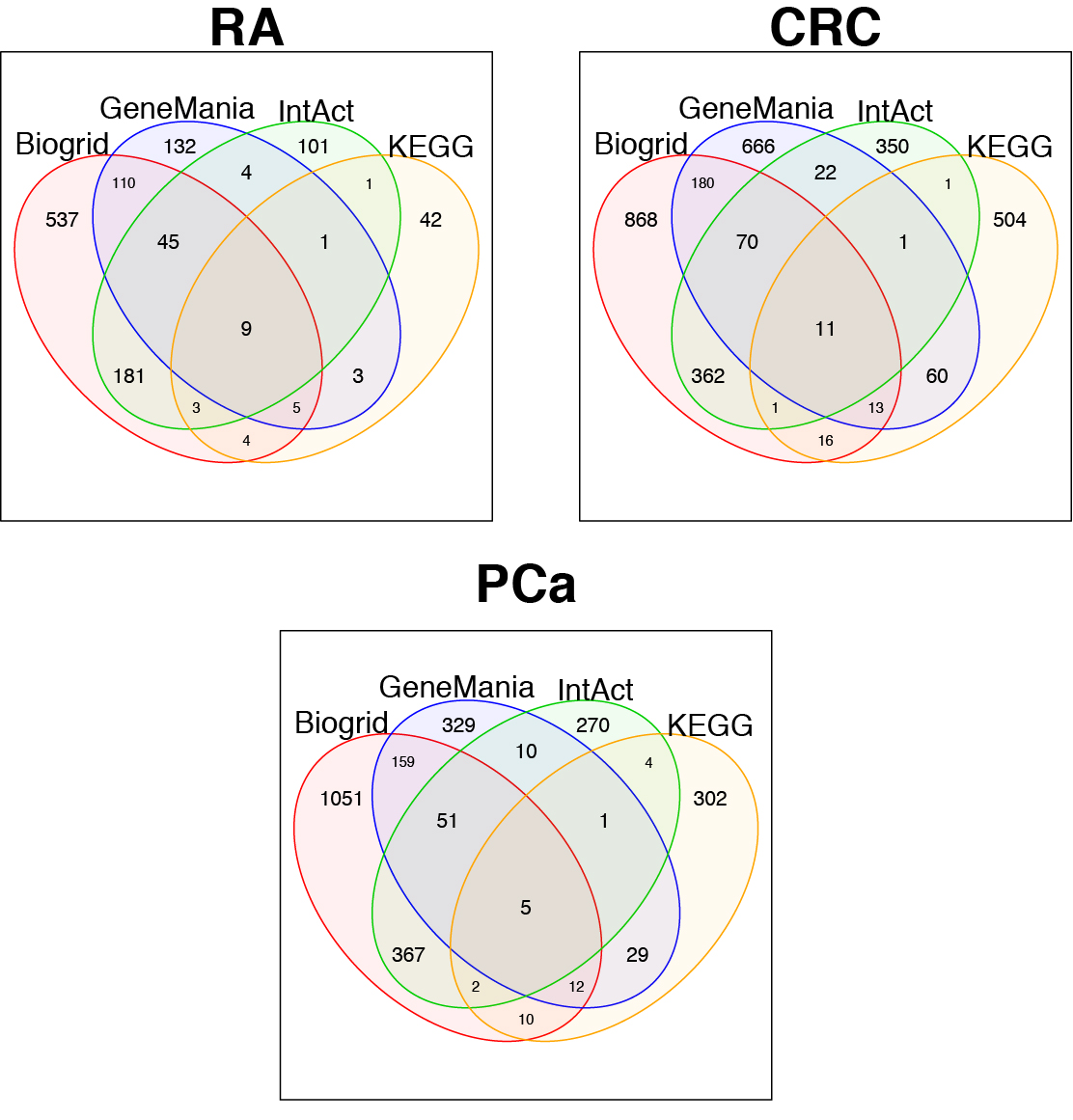

Supplement: Supplementary Figure 2 — Venn diagram of the numbers of direct interactions of input genes in each PIN. [file Image_2.jpeg]
