# Supplementary material for: pathfindR: An R Package for Comprehensive Identification of Enriched Pathways in Omics Data Through Active Subnetworks
Source: Front Genet. 2019 Sep 25;10:858. doi: 10.3389/fgene.2019.00858 (PMC6773876; doi:10.3389/fgene.2019.00858)
Supplement: Supplementary file 6 [file Table_1.docx]

**Supplementary Table 1. Common pathways between the CRC and PCa datasets.** ID indicates the KEGG id of the pathway, whereas Pathway indicates the pathway name. CRC_lowest_p and PCa_lowest_p indicate the lowest p-values for the CRC and PCa datasets, respectively.

| ID | Pathway | CRC_lowest_p | PCa_lowest_p |
| --- | --- | --- | --- |
| hsa05205 | Proteoglycans in cancer | 4.13869E-09 | 1.0385E-05 |
| hsa05130 | Pathogenic Escherichia coli infection | 8.18159E-09 | 0.012638019 |
| hsa04010 | MAPK signaling pathway | 1.20826E-08 | 6.38692E-06 |
| hsa04520 | Adherens junction | 1.80999E-08 | 8.70083E-05 |
| hsa04810 | Regulation of actin cytoskeleton | 2.16776E-08 | 6.58442E-05 |
| hsa04510 | Focal adhesion | 1.45627E-07 | 0.000548577 |
| hsa05012 | Parkinson disease | 7.90609E-07 | 0.005882045 |
| hsa04662 | B cell receptor signaling pathway | 1.44619E-06 | 0.015323796 |
| hsa04658 | Th1 and Th2 cell differentiation | 5.40366E-06 | 0.040720473 |
| hsa05165 | Human papillomavirus infection | 5.92132E-06 | 0.005787239 |
| hsa05161 | Hepatitis B | 6.4039E-06 | 0.031074222 |
| hsa04151 | PI3K-Akt signaling pathway | 7.98037E-06 | 0.000157043 |
| hsa04660 | T cell receptor signaling pathway | 8.66336E-06 | 0.007759806 |
| hsa04933 | AGE-RAGE signaling pathway in diabetic complications | 1.16834E-05 | 0.007170858 |
| hsa04625 | C-type lectin receptor signaling pathway | 2.16653E-05 | 5.66988E-06 |
| hsa05170 | Human immunodeficiency virus 1 infection | 6.40386E-05 | 0.00038872 |
| hsa04921 | Oxytocin signaling pathway | 6.74023E-05 | 0.000176674 |
| hsa05168 | Herpes simplex infection | 7.23269E-05 | 0.012818879 |
| hsa04022 | cGMP-PKG signaling pathway | 8.30192E-05 | 0.004947514 |
| hsa05132 | Salmonella infection | 0.000111497 | 0.001024926 |
| hsa04150 | mTOR signaling pathway | 0.00046493 | 0.03639603 |
| hsa05418 | Fluid shear stress and atherosclerosis | 0.000533585 | 0.016016719 |
| hsa05164 | Influenza A | 0.000671234 | 0.009418672 |
| hsa04014 | Ras signaling pathway | 0.001165472 | 0.000656891 |
| hsa05210 | Colorectal cancer | 0.00129243 | 0.007572536 |
| hsa05200 | Pathways in cancer | 0.001394025 | 0.001864931 |
| hsa04934 | Cushing syndrome | 0.002134647 | 0.012968007 |
| hsa04144 | Endocytosis | 0.00223191 | 0.000206535 |
| hsa04024 | cAMP signaling pathway | 0.002417989 | 0.035154932 |
| hsa04310 | Wnt signaling pathway | 0.002418113 | 0.000112173 |
| hsa05226 | Gastric cancer | 0.00267019 | 0.035466235 |
| hsa04392 | Hippo signaling pathway - multiple species | 0.002915258 | 1.03527E-07 |
| hsa04390 | Hippo signaling pathway | 0.003135632 | 1.35582E-05 |
| hsa04110 | Cell cycle | 0.003442925 | 0.00200072 |
| hsa05215 | Prostate cancer | 0.007778647 | 0.004678127 |
| hsa05160 | Hepatitis C | 0.010464676 | 0.012701709 |
| hsa04071 | Sphingolipid signaling pathway | 0.012088886 | 0.000468968 |
| hsa05016 | Huntington disease | 0.013246653 | 0.019575207 |
| hsa04270 | Vascular smooth muscle contraction | 0.014703261 | 2.48863E-05 |
| hsa05211 | Renal cell carcinoma | 0.017251888 | 0.001704596 |
| hsa05202 | Transcriptional misregulation in cancer | 0.017926078 | 0.002227785 |
| hsa04620 | Toll-like receptor signaling pathway | 0.023494048 | 0.002757387 |
| hsa04370 | VEGF signaling pathway | 0.025228423 | 0.043130708 |
| hsa05224 | Breast cancer | 0.028494806 | 0.002996465 |
| hsa04622 | RIG-I-like receptor signaling pathway | 0.030848585 | 0.036168176 |
| hsa04360 | Axon guidance | 0.032363714 | 0.000752109 |
| hsa04666 | Fc gamma R-mediated phagocytosis | 0.039418918 | 0.032172546 |
