# Supplementary material for: pathfindR: An R Package for Comprehensive Identification of Enriched Pathways in Omics Data Through Active Subnetworks
Source: Front Genet. 2019 Sep 25;10:858. doi: 10.3389/fgene.2019.00858 (PMC6773876; doi:10.3389/fgene.2019.00858)
Supplement: Supplementary file 7 [file Table_2.docx]

**Supplementary Table 2. Numbers of significantly enriched pathways (FDR < 0.05) for simple ORA analyses. “**all DEGs” indicate the numbers of significant pathways for analyses performed using all of the DEGs. **“**PIN DEGs” indicate the numbers of significant pathways for analyses performed using only DEGs found in the Biogrid PIN.

|  | RA | CRC | PCa |
| --- | --- | --- | --- |
| all DEGs | 0 | 11 | 4 |
| PIN DEGs | 1 | 22 | 8 |
