# Supplementary material for: pathfindR: An R Package for Comprehensive Identification of Enriched Pathways in Omics Data Through Active Subnetworks
Source: Front Genet. 2019 Sep 25;10:858. doi: 10.3389/fgene.2019.00858 (PMC6773876; doi:10.3389/fgene.2019.00858)
Supplement: Supplementary file 8 [file Table_3.docx]

**Supplementary Table 3. Comparison of interactions of PINs.**

| PIN A | PIN B | # of common interactions between PIN A and PIN B | % of interactions of PIN A also found in PIN B | % of interactions of PIN B also found in PIN A |
| --- | --- | --- | --- | --- |
| Biogrid | GeneMania | 35041 | 12.1% | 43.9% |
| Biogrid | IntAct | 68648 | 23.7% | 56.7% |
| Biogrid | KEGG | 4550 | 1.6% | 8.6% |
| GeneMania | IntAct | 14344 | 18% | 11.9% |
| GeneMania | KEGG | 6778 | 8.5% | 12.8% |
| IntAct | KEGG | 2676 | 2.2% | 5% |
